# Antioxidant and Anti-Inflammatory Effects of Genus *Gynura*: A Systematic Review

**DOI:** 10.3389/fphar.2020.504624

**Published:** 2020-11-27

**Authors:** Jiah Ning Tan, Shamin Mohd Saffian, Fhataheya Buang, Zakiah Jubri, Ibrahim Jantan, Khairana Husain, Norsyahida Mohd Fauzi

**Affiliations:** ^1^ Centre for Drug and Herbal Development, Faculty of Pharmacy, Universiti Kebangsaan Malaysia, Kuala Lumpur, Malaysia; ^2^ Centre for Quality Management of Medicines, Faculty of Pharmacy, Universiti Kebangsaan Malaysia, Kuala Lumpur, Malaysia; ^3^ Department of Biochemistry, Faculty of Medicine, Universiti Kebangsaan Malaysia, Kuala Lumpur, Malaysia; ^4^ Institute of Systems Biology, Universiti Kebangsaan Malaysia, Bangi, Malaysia

**Keywords:** *Gynura*, medicinal, plant, reactive oxygen species, antioxidant, anti-inflammatory

## Abstract

**Background:** *Gynura* species have been used traditionally to treat various ailments, such as fever, pain, and to control blood glucose level. This systematic review critically discusses studies regarding *Gynura* species that exhibited antioxidant and anti-inflammatory effects, thus providing perspectives and instructions for future research of the plants as a potential source of new dietary supplements or medicinal agents.

**Methods:** A literature search from internet databases of PubMed, Scopus, Science Direct, e-theses Online Service, and ProQuest was carried out using a combination of keywords such as “*Gynura*,” “antioxidant,” “anti-inflammatory,” or other related words. Research articles were included in this study if they were experimental (*in vitro* and *in vivo*) or clinical studies on the antioxidant or anti-inflammatory effects of *Gynura* species and if they were articles published in English.

**Results:** Altogether, 27 studies on antioxidant and anti-inflammatory effects of *Gynura* species were selected. The antioxidant effects of *Gynura* species were manifested by inhibition of reactive oxygen species production and lipid peroxidation, modulation of glutathione-related parameters, and enzymatic antioxidant production or activities. The anti-inflammatory effects of *Gynura* species were through the modulation of inflammatory cytokine production, inhibition of prostaglandin E_2_ and nitric oxide production, cellular inflammatory-related parameters, and inflammation in animal models. The potential anti-inflammatory signaling pathways modulated by *Gynura* species are glycogen synthase kinase-3, nuclear factor erythroid 2-related factor 2, PPARγ, MAPK, NF-κB, and PI3K/Akt. However, most reports on antioxidant and anti-inflammatory effects of the plants were on crude extracts, and the chemical constituents contributing to bioactivities were not clearly understood. There is a variation in quality of studies in terms of design, conduct, and interpretation, and in-depth studies on the underlying mechanisms involved in antioxidant and anti-inflammatory effects of the plants are in demand. Moreover, there is limited clinical study on antioxidant and anti-inflammatory effects of *Gynura* species.

**Conclusion:** This review highlighted antioxidant and anti-inflammatory effects of genus *Gynura* and supported their traditional uses to treat oxidative stress and inflammatory-related diseases. This review is expected to catalyze further studies on genus *Gynura*. However, extensive preclinical data need to be generated from toxicity and pharmacokinetic studies before clinical studies can be pursued for their development into clinical medicines to treat oxidative stress and inflammatory conditions.

## Introduction

Inflammation refers to a complex array of defensive immune responses (Arulselvan et al., 2016), and tissue damage is one of the consequences of an exaggerated or uncontrolled prolonged inflammatory process (Biswas, 2016). Inflammatory cells, including neutrophils and macrophages, generate free radicals at the inflammation site, where reactive oxygen species (ROS) (e.g., hydroxyl radicals, superoxide anion radicals, and hydrogen peroxide) act as both signaling molecules and inflammation mediators. Enhancement of pro-inflammatory gene expression can be achieved via the initiation of the intracellular signaling cascade by reactive species. The exaggerated generation of reactive species in pathological inflammatory conditions may induce localized oxidative stress and tissue injury, thus promoting progression of many inflammatory diseases (Mittal et al., 2014). Hence, inflammation and oxidative stress are highly interdependent pathophysiological events in various types of chronic diseases (Vaziri and Rodríguez-Iturbe, 2006; Biswas, 2016).

A total of 46 species are identified in the genus *Gynura* (The Plant List, 2020). They are distributed from tropical Africa to South and East Asia as well as Australia where the highest specific diversity is found in Southeast Asia (Vanijajiva and Kadereit, 2011). The fresh leaves of *G. procumbens* (Lour.) Merr. are traditionally consumed to control blood glucose level by Orang Asli in Kampung Bawong, Perak, West Malaysia (Samuel et al., 2010). *G. procumbens* is also traditionally used to treat kidney discomfort, inflammation, rheumatic fever, and viral ailments (Wiart, 2006). *G. pseudochina* (L.) DC. is traditionally used to treat fever and sore eye (Davies, 1980). In Chinese folk medicine, *G. segetum* (Lour.) Merr. is consumed as a decoction or is soaked in wine and orally taken to promote microcirculation or relieve pain (Chen et al., 2011). In Nepal, the juice of *G. nepalensis* DC. is applied on cuts and wounds as healing treatment (Manandhar, 2002).

Incorporation of a plant-based natural antioxidant in daily diet can prevent several human illnesses (Knekt et al., 1996) as the prevalence of many illness is inversely linked to the dietary consumption of antioxidant-rich foods (Sies, 1993). For any herb to be developed into a dietary supplement, it is essential that the evidence for the claimed effects of the herb is scientifically demonstrated. Ideally, the mechanisms of action should also be understood. The potential use of *Gynura* as a phytomedicine with antioxidant and anti-inflammatory properties has been well documented, but we were unable to identify specific review articles that focused on the antioxidant and anti-inflammatory effects of the *Gynura* species. This review was undertaken to assess published experimental data that investigated the antioxidant and anti-inflammatory activities of the *Gynura* species to support the design of future studies. It also provided an evaluation of the quality of available information, summarized mechanisms of action data from animal and cell studies following the administration of the *Gynura* species, and identified research gaps in the literature.

## Methods

This systematic review was carried out according to the Preferred Reporting Items for Systematic Reviews and Meta-Analyses (PRISMA) guideline (Moher et al., 2010). The quality of references was determined by referring to the Office Health Assessment and Translation for Conducting a Systematic Review and Evidence Integration (OHAT) guideline (Rooney et al., 2014).

### Search Strategy

The information was obtained through a comprehensive literature search using the electronic databases of PubMed, Scopus, and Science Direct from 2000 to March 2020 for journal articles and the databases of e-theses Online Service and ProQuest Dissertations and Theses Global for theses. The combination of keywords used in PubMed was as follows: [Gynura (Title/Abstract)] AND [antioxidant (Title/Abstract)] OR [oxidant (Title/Abstract)] OR [oxidative (Title/Abstract)] OR [oxidation (Title/Abstract)] OR [antiinflammatory (Title/Abstract)] OR [antiinflammation (Title/Abstract)] OR [inflammation (Title/Abstract)] OR [inflammatory (Title/Abstract)] OR [inflame (Title/Abstract)]. The reference lists of all included papers were checked for other potentially relevant citations. Studies selection was restricted to articles in English because of language barrier, time efficiency, and high cost for translation. However, only four studies were excluded on the basis of not being published in English, which is unlikely to impact our findings. In order to achieve a comprehensive search of relevant studies, university dissertations, and theses were accessed in the selection process. However, the confidentiality of these tools had possibly veiled some important information and results, for example, negative findings.

### Inclusion and Exclusion Criteria

Studies were included in this review if they were experimental studies (*in vitro* and *in vivo*) or clinical studies on the antioxidant or anti-inflammatory effects of *Gynura* and if they were articles published in English. The *Gynura* plant can be from any parts of the plant and in any form such as extracts, essential oils, fractions, or isolated compounds. Studies were excluded if they met at least one of the following criteria: 1) study models were not accepted as evidence for pharmacological effects (i.e., antioxidant experiments of FRAP, ABTS, DPPH, and Trolox equivalent antioxidant capacity assays), and 2) intervention was not focused on *Gynura*, although the antioxidant or anti-inflammatory effects were measured. Review articles and book chapters were excluded from this study, but the references were mined to search for further relevant studies.

### Data Extraction and Handling

The following details were extracted from each selected study: 1) the species and part of the *Gynura* used; 2) methods used, including animal species or cell lines, study design, and treatment details; 3) outcome measures; and 4) findings on the antioxidant and anti-inflammatory effects of *Gynura*.

### Quality Assessment

The reporting completeness of the material used by each selected study was assessed using the information of the *Gynura* species material, voucher specimens, report on quality control of extract and chemical analysis. The risk of bias in the included studies was assessed by two investigators based on the OHAT guideline for any potential bias such as selection bias, performance bias, attrition or exclusion bias, detection bias, selection reporting bias, and other sources of bias. However, some of the questions in the bias assessment were excluded as they were not applicable for use in the assessment of *in vitro*, *in vivo*, and human randomized control study designs.

## Results and Discussion

### Study Selection

**FIGURE 1:**
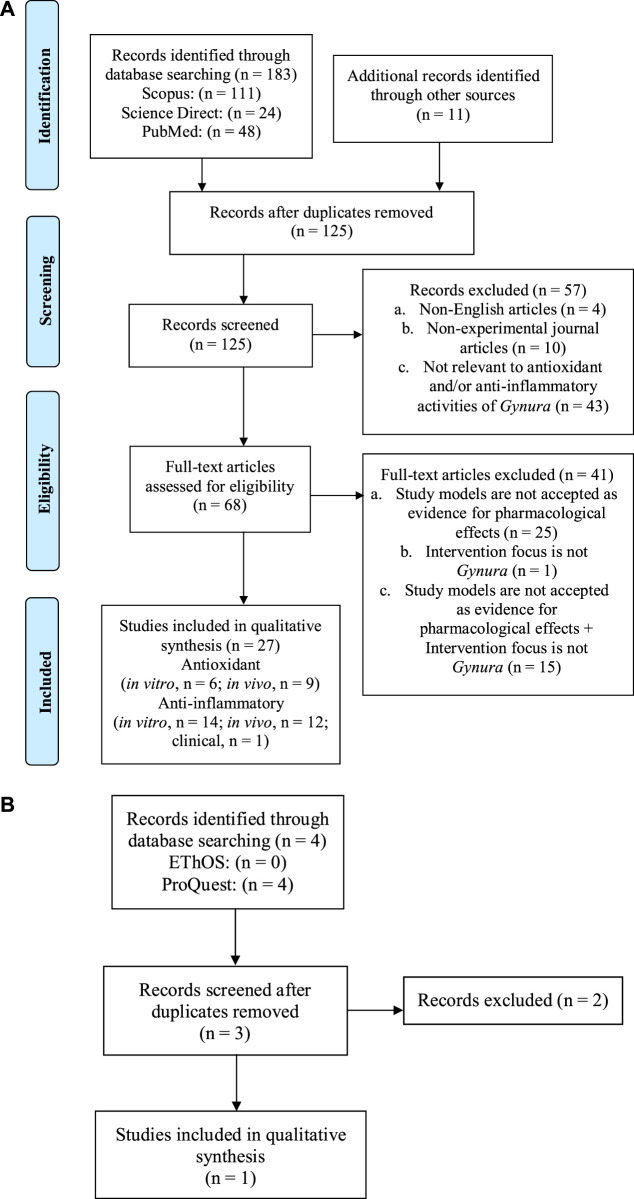
The flowchart shows the selection process of **(A)** the journal articles, **(B)** university dissertations, and theses in this systematic review based on the Preferred Reporting Items for Systematic Reviews and Meta-Analyses (PRISMA) guideline.

A total of 183 studies were found from the database search, and 11 additional articles were identified from other sources. After removing the duplicates, 125 articles were shortlisted, and after title–abstract screening, 57 articles were excluded because of the following reasons: The articles were published in languages other than English (n = 4), the articles are non-experimental journal articles (n = 10), and the articles reported on the effects of *Gynura* other than antioxidant and anti-inflammatory (n = 43). By full-text screening of the remaining 68 articles, a total of 41 articles were eliminated based on the following exclusion criteria: study models used are not accepted as evidence for pharmacological effects (n = 25), study intervention does not focus on *Gynura* (n = 1), and a combination of both exclusion criteria (n = 15). Thus, 27 articles from year 2002 to 2020 were selected in the final qualitative analysis of this systematic review. Three potential dissertations and theses were accessed through the two databases. Only one of the theses was selected, but the result presented in the theses has been published in one of the article selected in this review (Siriwatanametanon et al., 2010). Other two theses were excluded because they did not fulfill the inclusion criteria. A flowchart depicting the search process and study selection is presented in Figure 1.

### Quality Assessment

All selected articles were assessed for their quality and risk of bias. Table 1 shows the quality assessment of studies on the antioxidant and anti-inflammatory effects of *Gynura* species. The reporting completeness of the material in all selected studies was assessed using information of the *Gynura* species material, voucher specimens, quality control, and chemical analysis. Naming inconsistency between studies was detected as full botanical taxonomic names were not stated in 13 selected studies (Iskander et al., 2002; Siriwatanametanon and Heinrich, 2011; Akowuah et al., 2012; Seow et al., 2014; Chao et al., 2015; Wong et al., 2015; Xu et al., 2015; Rerknimitr et al., 2016; Yin et al., 2017; Rahman et al., 2018; Pai et al., 2019; Yang et al., 2019; Chandradevan et al., 2020). All plant species need to be validated taxonomically, and the full names have to be clearly stated as the incomplete name of plant species can lead to confusion of readers and any recorded uses or properties attributed may, in fact, correlate to different species. Such erroneous publications on the use of plant names are a permanent source of confusion for future research, search engines, and databases (Rivera et al., 2014). An incomplete plant name in some contexts may be ambiguous or may even mislead readers. This error is possible to occur within genus *Gynura. Gynura divaricata* is no guarantee to solely indicate *Gynura divaricata* (L.) DC. as *Gynura divaricata* subsp. *barbareifolia* (Gagnep.) F.G.Davies is a synonym of *Gynura barbareifolia* Gagnep and *Gynura divaricata* subsp. *formosana* (Kitam.) FG Davies is a synonym of *Gynura formosana* Kitam (The Plant List, 2020).

**TABLE 1:** Quality assessment of studies on the antioxidant and anti-inflammatory effects of *Gynura* species.

Study	Species stated in article	Plant source	Authenticated species	Quality control reported?	Chemical analysis reported?
Iskander et al. (2002)	*Gynura procumbens*	Department of Pharmacognosy, Faculty of Pharmacy, Mahidol University, Bangkok, Thailand	−	No	No
Akowuah et al. (2012)	*Gynura procumbens* (*Merr.,* compositae)	Penang Island, Malaysia	+	No	No
Kim et al. (2011)	*Gynura procumbens* (Lour.) Merr	Specialty Natural Products Co. Ltd., Thailand	+	No	Yes—HPLC, electrospray ionization time-of-flight mass spectrometry
Shwter et al. (2014)	*Gynura procumbens* (Lour.) Merr	Ethno Resources Sdn Bhd, Malaysia	+	No	No
Wong et al. (2015)	*Gynura procumbens*	Green House Facility, Faculty of Science and Technology, Universiti Kebangsaan Malaysia (UKM)	+	No	No
Huang et al. (2019)	*Gynura procumbens* (Lour.) Merr	Hainan Province, South China	+	No	Yes—GC-MS
Liu M. et al. (2019)	*Gynura procumbens* (Lour.) Merr	Jing’an, Jiangxi Province, China	−	No	Yes—ultra-high-performance liquid chromatography-quadrupole time-of-flight mass spectrometry
Nazri et al. (2019)	*Gynura procumbens* (Lour.) Merr	Semenyih, Selangor, Malaysia	+	No	Yes—LC-MS/MS
Ning et al. (2019)	*Gynura procumbens* (Lour.) Merr	Brightmark Sdn. Bhd, Semenyih, Malaysia	+	No	No
Liu Y. et al. (2019)	*Gynura procumbens* (Lour.) Merr	Shanxi Jintai Biol, China	−	No	Yes—LC-MS/MS
Chandradevan et al. (2020)	*Gynura procumbens*	Net house located in the Malaysia Agricultural, Research and development institute (MARDI)	+	No	Yes—^1^H NMR spectroscopy
Wu et al. (2013)	*Gynura bicolor* (Roxb. and Willd.) DC.	Yuanshan Village, Ilan, Taiwan	+	No	Yes—HPLC
Chao et al. (2015)	*Gynura bicolor* DC.	Farms in Puli Town, Nanton county, Taiwan	+	No	No
Yin et al. (2017)	*Gynura bicolor DC*	Farms in Puli Town, Nanton county, Taiwan	+	No	No
Pai et al. (2019)	*Gynura bicolor*	Farms, unknown location	−	No	No
Yang et al. (2019)	*Gynura bicolor* DC.	Farms, unknown location	−	No	No
Siriwatanametanon et al. (2010)	*Gynura pseudochina* (L.) DC. var. hispida Thv	Farmland in north-eastern part of Thailand, mainly in Buriram province	+	No	No
Siriwatanametanon and Heinrich (2011)	*Gynura pseudochina* (L.) var. hispida Thv	Not stated	−	No	Yes—electrospray ionization-mass spectrometry, ^1^H NMR, ^13^C NMR, 2D-NMR
Rerknimitr et al. (2016)	*Gynura pseudochina* DC. var. hispida Thv	Not stated	−	No	No
Sukadeetad et al. (2018)	*Gynura pseudochina* (L.) DC.	Koeng Sub-district, Mueang District, Maha Sarakham province, Thailand	+	No	Yes—HP-TLC, HPLC, LC-MS/MS
Seow et al. (2014)	*Gynura segetum*	Jabatan Pertanian Relau, Penang, Malaysia	+	No	No
Yuandani et al. (2017)	*Gynura segetum* (Lour.) Merr	Yogyakarta, West Java, Indonesia	+	No	Yes—HPLC
Yu et al. (2016)	*Gynura nepalensis* DC.	Suburb of Shanghai, China	+	No	Yes—HPLC
Rahman et al. (2018)	*Gynura nepalensis*	Kendua under Netrokona district of Bangladesh	+	No	Yes—method not stated
Xu et al. (2015)	*Gynura divaricata* L	Silk Biotechnology Laboratory, Soochow University, Suzhou, China	−	No	No
Dong et al. (2019)	*Gynura divaricata* (L.) DC.	Silk biotechnology Lab, Soochow University, Suzhou, China	−	No	Yes—HPLC
Ma et al. (2017)	*Gynura formosana* Kitam	Plant greenhouse of Longyan University, Longyan, China	−	No	No

HPLC, high-performance liquid chromatography; +, includes a voucher specimen; −, a voucher specimen is missing.

A total of 17 selected studies (Siriwatanametanon et al., 2010; Kim et al., 2011; Akowuah et al., 2012; Wu et al., 2013; Seow et al., 2014; Shwter et al., 2014; Chao et al., 2015; Wong et al., 2015; Yu et al., 2016; Yin et al., 2017; Yuandani et al., 2017; Rahman et al., 2018; Sukadeetad et al., 2018; Huang et al., 2019; Nazri et al., 2019; Ning et al., 2019; Chandradevan et al., 2020) provided the full information about the botanical material and authenticated the *Gynura* species by depositing voucher specimens. Six selected studies (Iskander et al., 2002; Xu et al., 2015; Ma et al., 2017; Dong et al., 2019; Liu M. et al., 2019; Liu Y. et al., 2019) reported the information of the *Gynura* species source but did not provide voucher specimens. Four selected studies (Siriwatanametanon and Heinrich, 2011; Rerknimitr et al., 2016; Pai et al., 2019; Yang et al., 2019) were inadequate on material information as the *Gynura* species source was not reported and voucher specimens were missing. The absence of voucher specimens seriously makes the reliability of the article suspicious. Sufficient description of the experimental methods used and citation of voucher specimens as evidence of the plants used are critical factors of the repeatability of the ethnopharmacological or any botanical study. Otherwise, the scientific impact of a study will be drastically diminished. Erroneous identification is a serious problem that may jeopardize any recorded uses or properties that may, in fact, relate to different species (Rivera et al., 2014).

All selected studies showed no data on quality control as none of the *Gynura* preparation was mentioned to follow the monograph of pharmacopeia. The safety, efficacy, and quality control of medicinal plants are getting more attention from both health authorities and the public. Herbal medicines are in widespread use, and the public believes that natural products are safe and devoid of adverse effects. However, medicinal plants are often used in combination, may be contaminated and adulterated, and may contain toxic compounds. The common misconception among the public often leads to inappropriate use and uncontrolled consumption, where poisoning and acute health problems are possible consequences. Hence, quality control of herbal medicines has a direct impact on their safety and efficacy (Wachtel-Galor and Benzie, 2011). According to good manufacturing practice, the crucial requirements for quality control of starting materials include correct identification of medicinal plant species, special storage, and special sanitation and cleaning methods for various materials (Ekor, 2014).

From harvesting to manufacturing, the quality of herbal medicines can be affected by vast factors. Quality evaluation of medicinal plants is possible by detecting the presence of chemical markers within a sample (Li et al., 2008). Qualitative chemical evaluation covers identification and characterization of phytochemical constituents in the medicinal plants through different analytical techniques. Phytochemical screening techniques involve botanical identification, extraction with suitable solvents, purification, and characterization of the active constituents of pharmaceutical importance (Folashade et al., 2012). Chromatographic methods such as thin-layer chromatography/high-performance TLC (TLC/HP-TLC) and high-performance liquid chromatography (HPLC), which are the most commonly used chemical techniques in the identification and quality assessment of medicinal plant ingredients, have provided characteristic qualitative and quantitative patterns of the constituents. Spectroscopic techniques, including UV, IR, and nuclear magnetic resonance (NMR), allow the quantitation of single or multiple compounds that share similarities in their UV absorbance, thus providing a more holistic view of herbal medicines in contrast to the quantitation of a single compound (Upton et al., 2019). Chemical analysis of *Gynura* species were reported in studies conducted by Kim et al. (2011), Siriwatanametanon and Heinrich (2011), Wu et al. (2013), Yu et al. (2016), Yuandani et al. (2017), Rahman et al. (2018), Sukadeetad et al. (2018), Dong et al. (2019), Huang et al. (2019), Liu M. et al. (2019), Liu Y. et al. (2019), Nazri et al. (2019), and Chandradevan et al. (2020) using methods such as HP-TLC, HPLC, gas chromatography-mass spectrometer, electrospray ionization-mass spectrometry, electrospray ionization time-of-flight mass spectrometry, liquid chromatography with tandem mass spectrometry, ultra-high-performance liquid chromatography-quadrupole time-of-flight mass spectrometry, ^1^H N^13^C NMR, and 2D-NMR.

On the basis of the risk of bias score in Table 2, under the domain “other sources of bias,” five studies (Siriwatanametanon et al., 2010; Kim et al., 2011; Siriwatanametanon and Heinrich, 2011; Chao et al., 2015; Rahman et al., 2018) were rated as “definitely high” risk of bias because of the unclear or absence of statistical analysis. This finding raised the validity of the interpretations of these studies in question because the claimed significant results are possibly misinterpreted under errors of statistical analysis. Hence, the erroneous statistical methods may contribute to false positive findings and may successively become misleading literature. Under the domain “Detection bias,” Iskander et al. (2002) and Rahman et al. (2018) were rated as definitely high risk of bias because the outcome assessment methods used were insensitive to indicate antioxidant or anti-inflammatory effects.

**TABLE 2:** Risk of bias assessment of each individual study on *Gynura* species according to OHAT guideline.

Domain	Questions	Iskander et al. (2002)	Akowuah et al. (2012)	Kim et al. (2011)	Shwter et al. (2014)	Wong et al. (2015)	Huang et al. (2019)	Liu M. et al. (2019)	Nazri et al. (2019)	Ning et al. (2019)	Liu Y. et al. (2019)	Chandradevan et al. (2020)	Wu et al. (2013)	Chao et al. (2015)	Yin et al. (2017)	Pai et al. (2019)	Yang et al. (2019)	Siriwatanametanon et al. (2010)	Siriwatanametanon and Heinrich (2011)	Rerknimitr et al. (2016)	Sukadeetad et al. (2018)	Seow et al. (2014)	Yuandani et al. (2017)	Yu et al. (2016)	Rahman et al. (2018)	Xu et al. (2015)	Dong et al. (2019	Ma et al. (2017)
Selection bias	1. Was administered dose or exposure level adequately randomized?	2	3	3	2	3	2	2	2	2	2	2	2	2	2	2	2	2	2	2	2	3	2	2	3	2	2	2
	2. Was allocation to study groups adequately concealed?	3	3	3	3	3	3	3	3	3	3	3	3	3	3	3	3	3	3	1	3	3	3	3	3	3	3	3
Performance bias	5. Were experimental conditions identical across study groups?	2	2	3	1	1	2	3	2	1	2	2	2	3	1	2	1	2	3	NR	1	1	3	2	2	2	2	3
	6. Were the research personnel and human subjects blinded to the study group during the study?	3	3	3	3	3	3	3	3	3	3	3	3	3	3	3	3	3	3	1	3	3	3	3	3	3	3	3
Attrition/exclusion bias	7. Were outcome data complete without attrition or exclusion from analysis?	3	3	3	3	3	3	3	3	3	3	3	3	3	3	3	3	3	3	3	3	3	3	3	3	3	3	3
Detection bias	8. Can we be confident in the exposure characterization?	3	3	3	3	3	3	3	3	3	3	3	3	3	3	3	3	3	3	3	3	3	3	3	3	3	3	3
	9. Can we be confident in the outcome assessment?	4	2	2	2	2	3	2	2	2	2	2	2	2	2	2	2	2	2	2	2	2	2	2	4	2	2	2
Selective reporting bias	10. Were all measured outcomes reported?	2	2	3	2	2	2	2	2	2	2	4	2	2	2	2	2	2	2	2	2	2	2	2	2	2	2	2
Other sources of bias	11. Were there no other potential threats to internal validity (e.g., statistical methods were appropriate and researchers adhered to the study protocol)?	3	1	4	1	2	2	2	1	1	1	2	1	4	1	1	1	4	4	2	1	1	2	1	4	1	2	1

^1^Definitely low risk of bias.

^2^Probably low risk of bias.

^3^or NR Probably high risk of bias or Not Reported.

^4^Definitely high risk of bias.

### Study Characteristics

#### Species of *Gynura*

Of the 27 selected studies, there are seven species of genus *Gynura*, which are *G. procumbens*, *G. bicolor*, *G segetum*, *G. divaricata*, *G. formosana*, *G. nepalensis*, and *G. pseudochina.* Among these *Gynura* species, 11 out of 27 selected studies focused on *G. procumbens* (Iskander et al., 2002; Kim et al., 2011; Akowuah et al., 2012; Shwter et al., 2014; Wong et al., 2015; Huang et al., 2019; Liu M. et al., 2019; Liu Y. et al., 2019; Nazri et al., 2019; Ning et al., 2019; Chandradevan et al., 2020), five studies on *G. bicolor* (Wu et al., 2013; Chao et al., 2015; Yin et al., 2017; Pai et al., 2019; Yang et al., 2019), four studies on *G. pseudochina* (Siriwatanametanon et al., 2010; Siriwatanametanon and Heinrich, 2011; Rerknimitr et al., 2016; Sukadeetad et al., 2018), two studies on *G. segetum* (Seow et al., 2014; Yuandani et al., 2017), two studies on *G. nepalensis* (Yu et al., 2016; Rahman et al., 2018), two studies on *G. divaricata* (Xu et al., 2015; Dong et al., 2019), and one study on *G. formosana* (Ma et al., 2017)*.*

Identical plant species might exhibit phytochemical or bioactivity variations that can possibly be attributed to intrinsic factors (age of the plant and part of the plant used) and extrinsic factors (geographical climate, nature of soil, season, and processing methods). The study conducted by Muraina et al. (2008) proved that the same plant extract from two different geographical locations varied in their phytochemical contents, cytotoxicity, antimicrobial and antioxidant activities. *G. bicolor* and *G. divaricata* extracts derived from plants originating from Nanjing were shown to possess higher free-radical-scavenging activities as compared to other same species extracts from different China origins (Chen et al., 2015). Hence, standardization and quality control of genus *Gynura* are crucial to gain regulatory approval of genus *Gynura* preparations as nutraceuticals or therapeutic drugs. Standardization of a drug can be used as a confirmation of its identity, quality, and purity throughout all phases of its cycle (Kumari and Kotecha, 2016). Stability testing and toxicity profiles of herbal products are important parameters in improving herbal product safety.

#### Part of Plant Used in Studies

Twenty selected studies used the leaves of *Gynura* to investigate their pharmacological effects. Nazri et al. (2019) and Ning et al. (2019) used the whole plant of *G. procumbens*, Xu et al. (2015) and Huang et al. (2019) used the leaf and stem parts of *Gynura*, Iskander et al. (2002) and Dong et al. (2019) used the aerial part of *Gynura*, and Liu M. et al. (2019) did not mention the part of *Gynura* used in their study. Hence, the leaf is the dominant part of the plant used in all selected research studies. Notably, the part of the plant that is usually consumed is the leaf. However, compared to the other parts of the plant, the root extract of both *G. procumbens* and *G. bicolor* exhibited the highest phenolic content, flavonoid content, ascorbic acid content, and antioxidant capacities (Krishnan et al., 2015). Mice with intraperitoneal injection of *G. procumbens* leaf extract at 25, 50, 100, and 250 mg/kg/day for four days survived until day 30 of post-injection with no signs of toxicity such as diarrhea, excess urination, and lethargy (Vejanan et al., 2012). On the basis of the study by Zahra et al. (2011) and Shwter et al. (2014), no mortality, or organ toxicity was detected in the animals that were orally administered *G. procumbens* leaf extract at doses of 2 and 5 g/kg for 14 days. The study carried out by Algariri et al. (2014) demonstrated that the leaf extract of *G. procumbens* was safe, with no observed acute toxicity effects as there was no treatment-related mortality at 2 g/kg throughout the 14 days observation period. Hence, the oral lethal dose (LD_50_) of *G. procumbens* extract for rats was determined to be greater than 2,000 mg/kg, and the acceptable daily intake was 700 mg/kg/day. The same study also showed that consumption of 250, 500, and 1,000 mg/kg of *G. procumbens* extract for 28 days did not lead to sub-chronic toxicity as no abnormal behavior, disease, or death was observed in the animal model that received the plant extract. Administration of *G. procumbens* leaf extract at 1,000–5,000 mg/kg did not cause mortality or significant changes in the general behavior, bodyweight, or organ gross appearance of rats (Rosidah et al., 2009). At the same time, *G. procumbens* and *G. bicolor* leaf extracts both showed a negligible level of toxicity when administered orally at 300, 2,000, and 5,000 mg/kg for 14 days (Teoh et al., 2013, 2016).

However, the use of genus *Gynura* requires extra caution on toxic risk because of the presence of hepatotoxic pyrrolizidine alkaloids which was determined in the leaves of *G. pseudochina* (Siriwatanametanon and Heinrich, 2011; Sukadeetad et al., 2018) as well as the aerial part of *G. bicolor* and *G. divaricata* (Chen et al., 2017). Hepatic injury caused by genus *Gynura* has been reported in few other studies. By daily administration of the root of *G. segetum* at 1.0 g extract/kg for 40 successive days, hepatic veno-occlusive disease, also called hepatic sinusoidal obstruction syndrome (HSOS), was induced in the mouse model where liver fibrosis-related factors and pro-inflammatory cytokines were upregulated (Zhang et al., 2019). The mouse model in which a 30 g/kg decoction of *G. segetum* dried rhizome was administered by gavage in the morning for 30 days showed features of HSOS, including increased weight ratio of the liver and body, serum transaminase, bilirubin, decreased albumin (Chen et al., 2011), bulging abdomen, a large number of clear ascites, floating bowels, jelly omentum in the abdominal cavity, and congested and swollen or contracted and hard liver with a grainy surface and blunt edges (Zhu et al., 2011). The potential markers of hepatotoxicity induced by the decoction of *G. segetum* dried rhizome are verified in the animal model as differential metabolites of arginine, creatine, valine, glutamine, and citric acid, which are involved in the regulation of multiple metabolic pathways, primarily amino acid metabolism and energy metabolism (Qiu S. et al., 2018). *G. segetum* dried rhizome also caused liver injury by dysregulating mitochondrial ROS generation through the SIRT3-SOD2 pathway (Li et al., 2019). The roots and aerial parts of *G. segetum* were proven to contain large amounts of pyrrolizidine alkaloids, including senecionine, which were responsible for the impaired bile acid homeostasis in the animal model orally administered 1.0 g/kg BW *G. segetum* extract for 48 h (Xiong et al., 2018). Hence, any contraindications caused by long-term consumption of genus *Gynura* must be determined prior to the commercialization of genus *Gynura* products. Appropriate clinical studies are needed to determine the optimal efficacy and minimum toxicity of genus *Gynura*. The application of genus *Gynura* must be closely monitored in term of doses and qualities, especially the species with reported cases of hepatotoxicity.

#### Solvents Used for Extraction

Five studies used the methanol extracts of *G. procumbens, G. pseudochina* and *G. segetum* to investigate their antioxidant or anti-inflammatory activities in HeLa cells (Siriwatanametanon et al., 2010), human leukocytes (Siriwatanametanon et al., 2010; Yuandani et al., 2017), murine macrophages (Yuandani et al., 2017), oxidative stress model rats (Akowuah et al., 2012), and granuloma model rats (Seow et al., 2014). As a continuation study of Siriwatanametanon et al. (2010), Siriwatanametanon and Heinrich (2011) as well as Yuandani et al. (2017) isolated compounds from the methanol extract of *Gynura* and determined their antioxidant or anti-inflammatory effects in HeLa cells or macrophages. The extraction of *G. procumbens* with 80% methanol at a temperature below 60°C would give greater retention of the total phenolic content (TPC) and greater expression of free radical scavenging activity (FRSA) (Akowuah et al., 2009). Methanol was also identified as a more effective extraction solvent of *G. procumbens* compared to 95% ethanol and water extracts based on its higher TPC and FRSA (Akowuah et al., 2012). Sukadeetad et al. (2018) demonstrated a higher chlorogenic acid content in *G. pseudochina* leaf extract by microwave drying as well as good efficiency for recovering phenolic compounds by extraction with 50% (v/v) methanol. However, ethanol extract was used in this study for successive bioassays by considering ethanol as a safer solvent for health product application.

Eleven studies tested the antioxidant or anti-inflammatory activities of the ethanol extract of *G. procumbens, G. bicolor, G. pseudochina* and *G. nepalensis* in human keratinocytes (Kim et al., 2011; Sukadeetad et al., 2018), human dermal fibroblasts (Kim et al., 2011), human endothelial cells (Chao et al., 2015), murine macrophages (Chandradevan et al., 2020; Liu M. et al., 2019; Ning et al., 2019), ear-inflamed mice (Iskander et al., 2002; Rahman et al., 2018), rat colon cancer model (Shwter et al., 2014), parasite-infected mice (Wong et al., 2015), and hypercholesterolemic rats (Nazri et al., 2019). Liu M. et al. (2019) simultaneously studied the anti-inflammatory effect of the ethanol extract and its fractions, including the petroleum ether fraction, ethyl acetate fraction, *n-*butanol fraction, and water fraction. Yu et al. (2016) studied the antioxidant and anti-inflammatory effects of nine caffeoylquinic acid analogs isolated from the ethanol extract on cardiomyoblasts. The optimal extraction of *G. divaricata* was determined with 45% ethanol for 30 min at 90°C, where the increase in the extraction temperature (from 40 to 100°C) led to a significant elevation of the TFC, TPC, and FRSA (Wan et al., 2011). Another study on the extraction method optimization of *G. bicolor* showed that 40% ethanol, 40°C, 30 min sonication time, and 50:1 liquid-to-solid ratio were the optimal conditions for a higher extraction yield of TPC (Qiu X. L. et al., 2018).

Five studies used the aqueous extract of *G. procumbens* and *G. bicolor* to determine their antioxidant or anti-inflammatory activities in human endothelial cells (Chao et al., 2015), murine adrenal gland pheochromocytoma (Yang et al., 2019), murine hepatocytes (Liu Y. et al., 2019), diabetic mice (Pai et al., 2019), and liver-injured mice (Yin et al., 2017; Liu Y. et al., 2019). The ethyl acetate extract of *G. formosana* was studied for its antioxidant and anti-inflammatory activities in granuloma model rats (Ma et al., 2017). Meanwhile, the ether extract of *G. bicolor* was studied for its anti-inflammatory activity in murine macrophages (Wu et al., 2013). *G. divaricata* was lyophilized into powder and tested in diabetic mice (Xu et al., 2015; Dong et al., 2019). The study by Huang et al. (2019) determined the anti-inflammatory effect of *G. procumbens* essential oil and its active ingredients in murine macrophage and ear-inflamed mice. One randomized controlled study on patients with moderate plaque psoriasis was conducted to study the anti-inflammatory effect of *G. pseudochina* ointment from an ethanol extract with a ratio of one extract to 10 vehicles (Rerknimitr et al., 2016).

#### Antioxidant Parameters

In general, ROS production level was the major parameter measured *in vitro* antioxidant studies (Kim et al., 2011; Chao et al., 2015; Yu et al., 2016; Yuandani et al., 2017; Liu Y. et al., 2019; Yang et al., 2019). The other *in vitro* parameters included glutathione (GSH) content and GSH peroxidase (GSH-Px) or catalase (CAT) activity (Chao et al., 2015; Yu et al., 2016; Yang et al., 2019). The main focus of *in vivo* antioxidant studies of *Gynura* species was lipid peroxidation (Akowuah et al., 2012; Shwter et al., 2014; Xu et al., 2015; Ma et al., 2017; Dong et al., 2019; Liu Y. et al., 2019; Nazri et al., 2019). The other *in vivo* parameters measured were plasma total antioxidant status (TAS) (Akowuah et al., 2012), CAT activity (Ma et al., 2017; Liu Y. et al., 2019; Nazri et al., 2019; Pai et al., 2019), ROS production (Yin et al., 2017; Pai et al., 2019), superoxide dismutase (SOD) activity (Shwter et al., 2014; Xu et al., 2015; Ma et al., 2017; Dong et al., 2019; Nazri et al., 2019), heme oxygenase 1 (HO-1) (Liu Y. et al., 2019), 8-hydroxy-2′-deoxyguanosine (8-OHdG) level (Dong et al., 2019), and GSH-related parameters, including GSH-Px activity (Xu et al., 2015; Yin et al., 2017; Dong et al., 2019; Liu Y. et al., 2019; Nazri et al., 2019; Pai et al., 2019), GSH-*S*-transferase (GST) activity (Shwter et al., 2014), GSH reductase (GR) activity (Yin et al., 2017; Pai et al., 2019), GSH disulfide (GSSG) content (Yin et al., 2017), and GSH content (Ma et al., 2017; Yin et al., 2017; Pai et al., 2019).

#### Anti-inflammatory Parameters

Overall, *in vitro* anti-inflammatory studies on *Gynura* species focused on the secretion or expression of pro-inflammatory mediators, including interleukin-1 (IL-1) (Siriwatanametanon et al., 2010; Yuandani et al., 2017; Yang et al., 2019), IL-6 (Siriwatanametanon et al., 2010; Kim et al., 2011; Chao et al., 2015; Liu M. et al., 2019; Yang et al., 2019), IL-8 (Kim et al., 2011; Sukadeetad et al., 2018), tumor necrosis factor alpha (TNF-α) (Siriwatanametanon et al., 2010; Chao et al., 2015; Yuandani et al., 2017; Liu M. et al., 2019; Yang et al., 2019), prostaglandin E_2_ (PGE_2_) (Siriwatanametanon et al., 2010; Wu et al., 2013; Chao et al., 2015), cyclooxygenase-2 (COX-2) (Wu et al., 2013; Chao et al., 2015), inducible nitric oxide synthase (iNOS) (Wu et al., 2013; Ning et al., 2019), nitric oxide (NO) (Wu et al., 2013; Yuandani et al., 2017; Liu M. et al., 2019; Ning et al., 2019; Chandradevan et al., 2020), lactate dehydrogenase (LDH) activity, mitochondrial membrane potential (Δψm) (Yu et al., 2016; Yang et al., 2019), matrix metalloproteinase-1 and 9 (MMP-1 and MMP-9) (Kim et al., 2011), and infiltration of inflammatory cells (Huang et al., 2019). Moreover, *in vitro* studies included investigations on the anti-inflammatory effect of the *Gynura* species on the nuclear factor erythroid 2-related factor 2 (Nrf2) signaling pathway (Liu Y. et al., 2019), nuclear factor kappa B (NF-κB) signaling pathway (Siriwatanametanon et al., 2010; Siriwatanametanon and Heinrich, 2011; Wu et al., 2013; Sukadeetad et al., 2018; Yang et al., 2019), and mitogen-activated protein kinases (MAPK) signaling pathway, including c-Jun N-terminal kinase (JNK) (Yu et al., 2016; Liu Y. et al., 2019), p38 (Yu et al., 2016; Yang et al., 2019), and extracellular signal-regulated kinase (ERK) (Yu et al., 2016). Similarly, the *in vivo* studies also measured the parameters of IL-1 (Seow et al., 2014; Ma et al., 2017; Yin et al., 2017; Pai et al., 2019), IL-6 (Yin et al., 2017; Pai et al., 2019), and TNF-α (Seow et al., 2014; Wong et al., 2015; Ma et al., 2017; Yin et al., 2017; Dong et al., 2019; Pai et al., 2019). The other anti-inflammatory effect parameters of *in vivo* studies were interferon-γ (IFN-γ), IL-10, phosphorylation of glycogen synthase kinase-3 (GSK3β (Wong et al., 2015; Dong et al., 2019), LDH, alanine aminotransferase (GPT), c-reactive protein (CRP) (Ma et al., 2017), Nrf2 (Liu Y. et al., 2019), peroxisome proliferator-activated receptor gamma (PPARγ) (Dong et al., 2019; Liu Y. et al., 2019), COX-2 (Huang et al., 2019), inflamed-ear thickness (Iskander et al., 2002; Rahman et al., 2018; Huang et al., 2019), cotton pellet granuloma (Seow et al., 2014; Ma et al., 2017), paw edema (Rahman et al., 2018; Huang et al., 2019), signaling pathways of phosphatidylinositol 3-kinase (PI3K)/protein kinase B (Akt) (Xu et al., 2015; Dong et al., 2019), JNK (Liu Y. et al., 2019), p38 (Pai et al., 2019), and NF-κB (Dong et al., 2019; Pai et al., 2019). The effect of *Gynura* on NF-κB phosphorylation was also studied in a randomized controlled study (Rerknimitr et al., 2016).

### Antioxidant Effects of Genus *Gynura*

**FIGURE 2:**
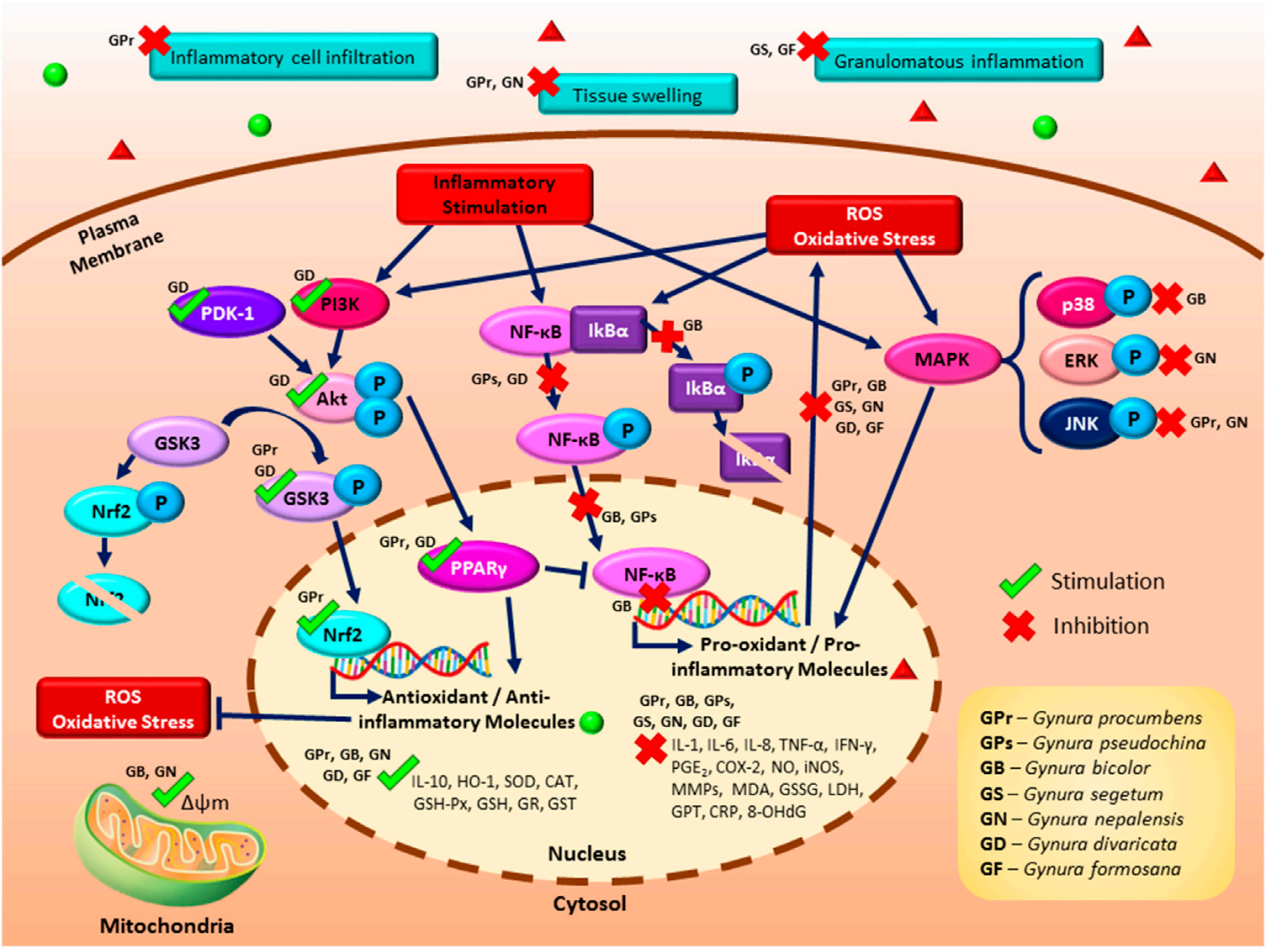
A schematic illustrating the proposed signaling pathways of the antioxidant and anti-inflammatory effects of *Gynura* species. The antioxidant effects of *Gynura* are manifested by the inhibition of reactive oxygen species (ROS) production, suppression of lipid peroxidation, modulation of enzymatic antioxidant production or activities, and modulation of glutathione-related parameters. The reported anti-inflammatory effects of *Gynura* are modulation of inflammatory cytokines and molecule production as well as inhibition of cellular inflammation and inflammation in an animal model. The potential anti-inflammatory signaling pathways of *Gynura* include the induction of the phosphatidylinositol 3-kinase (PI3K)/protein kinase B (Akt), nuclear factor erythroid 2-related factor 2 (Nrf2), and peroxisome proliferator-activated receptor gamma (PPARγ) signaling pathways as well as the inhibition of the nuclear factor kappa B (NF-κB), glycogen synthase kinase 3 (GSK3), and mitogen-activated protein kinase (MAPK) signaling pathways. Dark blue arrow lines indicate activation, dark blue perpendicular lines indicate inhibition, green tick indicates induction by *Gynura* species, and red cross indicates suppression by *Gynura* species.

Table 3 shows the list of studies on the antioxidant effects of genus *Gynura.* Several potential mechanisms for the antioxidant activity of *Gynura* are suggested as follows: inhibition of ROS, inhibition of lipid peroxidation, modulation of enzymatic antioxidant production or activities, and modulation of GSH-related parameters. Figure 2 illustrates the proposed signaling pathways of antioxidant effects by *Gynura* species.

**TABLE 3:** List of studies on the antioxidant effects of genus *Gynura.*

Plant species	Part, *Gynura* form	Cell line/Animal study model	Concentration/dose, control groups	Parameter measured and technique used	Findings	Reference
*Gynura procumbens* (lour.) Merr	Leaf	Human HaCaT keratinocytes	1, 10, 50 μg/ml	Intracellular ROS production level by dichlorofluorescein (DCF) content	GP extract treatment inhibited UV-induced ROS generation levels about 36% at 50 μg/ml	Kim et al. (2011)
			Control groups			
			Normal control			
			Model control (UV 40 mJ/cm^2^)			
			Reference group (50, 100, 200 μg/ml vitamin C)			
	Extract (ethanol)					
		*in vitro* study stimulated by UV irradiation				
	Leaf	Mice normal liver cell line NCTC-1469	80 and 160 μg/ml (24 h)	Intracellular ROS production level	↓ ROS level by 80 and 160 μg/ml GP extract	Liu Y. et al. (2019)
			Control groups			
			Normal control (culture medium)			
	Extract (aqueous)					
			Model control (pre-treated with 0.25 mM palmitic acid (PA) + 0.5 mM oleic acid for 24 h)			
		*In vitro* study stimulated by palmitic acid and oleic acid				
	Leaf	18 sprague-dawley rats (6 rats/group)	1.0 g/kg body weight	Plasma lipid peroxidation levels using thiobarbituric acid reactive substances (TBARS) assay	↓ plasma TBARS level	Akowuah et al. (2012)
			Oral route			
	Extract (methanol)	*In vivo* study using carbon tetrachloride (CCl_4_)-induced oxidative stress rats	Daily single dose for 14 days			
					TAS values of rats fed with the extract (1 g/kg) for 14 days followed by CCl_4_ administration were comparable to values of control group	
			Control groups	Plasma total antioxidant status (TAS)		
			Normal control			
			Model control (single dose of CCl_4_)			
	Leaf	30 adult male sprague-dawley rats (6 rats/group)	250 and 500 mg/kg body weight	Lipid peroxidation levels using TBARS assay	↑ GST and SOD activities in treated rats (250 and 500 mg/kg)	Shwter et al. (2014)
			Oral route			
			Daily for 10 weeks	Glutathione-S-transferase (GST) activity		
				Superoxide dismutase (SOD) activity		
			Control groups			
			Normal control (normal saline subcutaneous injections, 10% tween 20 oral administration)			
		*In vivo* study using carcinogen-induced colon cancer rats				
					↓ MDA level in treatment groups 250 and 500 mg/kg GP.	
			Carcinogen group (azoxymethane (AOM) subcutaneous injection +10% tween 20 oral administration)			
			Reference group (AOM, fluorouracil intraperitoneal injection)			
	Extract (ethanol)					
	Whole plant	48 female sprague-dawley rats (6 rats/group)	250 and 500 mg/kg body weight	Plasma malondialdehyde (MDA) level using high-performance liquid chromatography (HPLC)	↓ MDA level in treatment groups 250 and 500 mg/kg GP compared to postmenopausal control group at month 3 and month 6	Nazri et al. (2019)
					↑ SOD, GSH-Px and CAT enzyme activities in treated rats (250 and 500 mg/kg) compared to postmenopausal control group. GP maintained SOD activity from month 3 to month 6	
			Oral route			
	Extract (ethanol)					
			Daily for 24 weeks			
		*In vivo* study using postmenopausal rats fed with cholesterol diet enriched with repeatedly heated palm oil		Superoxide dismutase (SOD) activity		
				Glutathione peroxidase (GSH-Px) activity		
			Control groups			
			Sham group (rats received basal diet)			
			Postmenopausal (PM) group (rats subjected to ovariectomy, received 2% cholesterol diet fortified with five-time heated palm oil (5HPO)			
				catalase (CAT) activity		
			Positive control (10 mg/kg atorvastatin)			
	Leaf	32 male C57BL/6 J mice (8 mice/group)	500 and 1,000 mg/kg body weight	Hepatic malondialdehyde (MDA) level	↓↓ MDA level in treatment groups 500 and 1,000 mg/kg GP.	Liu Y. et al. (2019)
	Extract (aqueous)		Oral route			
				Glutathione peroxidase (GSH-Px) activity		
		*In vivo* study using non-alcoholic steatohepatitis (NASH) mice				
					↑↑ GSH-Px, CAT and HO-1 activities in treated mice (500 and 1,000 mg/kg)	
			Daily for 6 weeks			
				catalase (CAT) activity		
			Control groups			
				Heme oxygenase 1 (HO-1) activity		
			Normal control (methionine- and choline-sufficient (MCS) diet)			
			Model control (methionine- and choline-deficient (MCD) diet)			
*Gynura bicolor* (roxb. Ex willd.) DC.	Leaf	Human umbilical vein endothelial cells	Aqueous or ethanol extract at 1, 2 or 4% (v/v)	ROS production level by 2′,7′-dichlorofluorescein (DCF) content	Pre-treatments with aqueous or ethanol extract dose-dependently ↓ ROS level and preserved GSH content	Chao et al. (2015)
	Extract (aqueous, ethanol)					
			12 h pre-treatment			
				catalase (CAT) activity		
					Pre-treatment at 2 and 4% retained CAT and GSH-Px activities	
		*In vitro* study treated by high glucose				
			Control groups			
				Glutathione (GSH) content		
			Control (5.5 mM glucose)			
				Glutathione peroxidase activity (GSH-Px)		
			Model control (33 mM glucose)			
	Leaf	PC12 cell line (rat adrenal gland pheochromocytoma)	Aqueous extract at 0.25, 0.5 or 1%	ROS level by 2′,7′-Dichlorofluorescein diacetate (DCFH-DA)	GB pre-treatments ↑ GSH content, ↑ GSH-Px activity, concentration-dependently ↓ ROS level and ↑ catalase activity	Yang et al. (2019)
			48 h pre-treatment			
				Glutathione level (GSH)		
	Extract (aqueous)					
			Control groups			
				Glutathione peroxidase activity (GSH-Px)		
			Normal control	CAT activity assay		
			Model control (H_2_O_2_ stimulation)			
		*In vitro* study of H_2_O_2_ induced injury				
	Leaf	Male C57BL/6 mice (8 mice/group)	0.25 or 0.5% *G. bicolor* aqueous extract diet	Hepatic GSH or GSSG content	0.25% or 0.5% GB ↑ GSH content, ↓ GSSG, ↓ ROS levels, maintained GSH-Px, GR, and catalase activities	Yin et al. (2017)
	Extract (aqueous)					
		*In vivo* study using chronic ethanol consumption-induced hepatic injury mice model				
				Glutathione peroxidase activity (GSH-Px)		
			Oral route			
				Glutathione reductase (GR) and catalase		
			6 weeks			
			Control groups			
				Hepatic ROS level using DCFH-DA		
			Normal control			
			Liquid diet group (without ethanol)			
			Ethanol diet group			
vivoA	Leaf	50 male Balb/cA mice (10 mice/group)	0.25, 0.5, 1% GB diet	ROS level by DCFH-DA	GB at 3 doses	Pai et al. (2019)
			Oral route	Glutathione level (GSH)		
			8 weeks			
					↑ GSH content, ↑ GPX, ↑ GR, and ↑ catalase activities↓ ROS level in heart and kidney	
				Glutathione peroxidase activity (GSH-Px)		
			Control groups			
	Extract (aqueous)					
				Glutathione reductase (GR)		
			Normal control (standard mouse basal diet)			
				CAT activity assay		
			Diabetic model control (40 mg/kg BW streptozotocin via i.p. injection for 5 days)			
		*In vivo* streptozotocin-induced type 1 diabetic mice model				
*Gynura divaricata* (L.) DC.	Leaf, stem	60 male imprinting control region mice (15 mice/group)	Diets with 1.2% and 4.8% GD	Glutathione peroxidase activity (GSH-Px)	1.2% GD ↑↑ T-SOD levels, ↓↓	Xu et al. (2015)
			Oral route		MDA level 4.8% GD	
	Lyophilized into powder			Total superoxide dismutase activity (SOD)		
					↑↑ GSH-Px level, ↑ T-SOD level, ↓ MDA level	
				Lipid peroxidation level by MDA level assay		
		*In vivo* study using high-fat diet and streptozotocin (STZ) induced type 2 diabetic mice				
			Daily for 4 weeks			
			Control groups			
			Normal control (normal diet)			

			Diabetic model control (high-fat diet (18% lard, 20% sugar, 3% egg yolk, 59% basal diet) and 100 mg/kg STZ)			
	Aerial part	Male ICR mice (15 mice/group)	Diets with 1%, 5% and 10% GD	Hepatic glutathione peroxidase activity (GSH-Px)	1% GD: ↑ GSH-Px level, ↑ T-SOD level	Dong et al., (2019)
	Lyophilized into powder					
		*In vivo* study using high-fat diet and streptozotocin (STZ) induced type 2 diabetic mice	Oral route			
					5% GD: ↑ GSH-Px level, ↑↑ T-SOD level, ↓ 8-OHdG level	
			Daily for 4 weeks	Hepatic total superoxide dismutase activity (SOD)		
				Hepatic lipid peroxidation level by MDA level assay		
					10% GD: ↑↑ GSH-Px level, ↑ T-SOD level, ↓ MDA level, ↓ 8-OHdG level	
			Control groups			
			Normal control (normal chow)			
				Hepatic 8-hydroxy-2′-deoxyguanosine (8-OHdG) level using ELISA		
			Model control (high-fat diet (18% lard, 20% sugar, 3% egg yolk and 59% basal diet) and 100 mg/kg STZ)			
*Gynura segetum* (lour.) Merr	Leaf	Polymorphonuclear cells neutrophils (PMN)	Extract: 6.25–100 μg/ml	ROS production level by luminol method	GS extract exhibited inhibitory activity upon activation by PMA (IC_50_ = 1.41 ± 0.63 μg/ml) and zymosan (IC_50_ = 2.63 ± 0.89 μg/ml)	Yuandani et al. (2017)
			Compounds: 3.125–50 μg/ml			
			Control groups			
			Negative control (without sample)			
					8,8′-(ethene-1,2-diyl)-dinaphtalene-1,4,5-triol revealed ROS inhibitory upon activation by PMA (IC_50_ = 0.13 μM) and zymosan (IC_50_ = 0.05 μM)	
	Extract (methanol), isolated compounds from extract					
		*In vitro* study induced by opsonized zymosan or PMA				
			Positive control (acetylsalicylic acid)			
					Rutin inhibited ROS activated by PMA (IC_50_ = 0.08 μM) and zymosan (IC_50_ = 0.13 μM)	
*Gynura nepalensis* DC.	Leaf	H9c2 cardiomyoblasts	0.78, 1.56, 3.12, 6.25, 12.5, 25, 50, 100 μM	Intracellular ROS production by DCF content	Compound 6 (3,5-dicaffeoylquinic acid ethyl ester) exhibited a more potent cytoprotective effect thus selected for further evaluation	Yu et al. (2016)
			1 h pre-treatment			
			Control groups			
		*In vitro* study stimulated by H_2_O_2_				
	Nine caffeoylquinic acid analogs (1–9) isolated from ethanol extract					
				CAT activity assay		
					↓↓↓ ROS production in H_2_O_2_-treated cells, even at a concentration of 0.78 μM	
			Normal control			
			Model control (0.3 mM H_2_O_2_)			
					↑↑↑ H_2_O_2_-induced decrease in CAT activity at doses of 25 and 50 μM	
*Gynura formosana* kitam	Leaf	48 male sprague-dawley rats (8 rats/group)	100 mg/kg, 250 mg/kg, 500 mg/kg body weight	CAT activity assay	250 and 500 mg/kg GF treatment ↑↑ activities of CAT, ↑↑ SOD, ↑↑ GSH and ↓↓ lipid peroxidation in rat liver	Ma et al. (2017)
				Total superoxide dismutase activity (SOD)		
			Oral route			
		*In vivo* study using cotton pellet-induced granuloma rat model	Once daily for 7 days	Glutathione level (GSH)		
				Lipid peroxidation level		
			Control groups			
	Extract (ethyl acetate)					
			Normal control			
			Model control (0.5% carboxymethylcellulose, 1 ml/kg)			
			Standard drug (4 mg/kg indomethacin)			

↑ indicates significantly induce (*p* < 0.05), ↑↑ indicates significantly induce (*p* < 0.01), ↑↑↑ indicates significantly induce (*p* < 0.001), ↓ indicates significantly inhibit (*p* < 0.05), ↓↓ indicates significantly inhibit (*p* < 0.01), and ↓↓↓ indicates significantly inhibit (*p* < 0.001).

#### *Gynura* Inhibits Reactive Oxygen Species

At physiological concentrations, ROS is involved in many cellular activities, including gene transcription, signaling transduction, and immune response. However, overproduction of ROS can result in oxidative damage to biomolecules, including lipids, proteins, and DNA, which is the underlying cause of various diseases (Liu et al., 2018). Inhibition of ROS production using *G. procumbens* extracts was determined in human HaCaT keratinocytes at 50 μg/ml (inhibitory effect comparable to 200 μg/ml vitamin C) (Kim et al., 2011) and in murine hepatocytes at 80 and 160 μg/ml (Liu Y. et al., 2019); that of *G. bicolor* in human umbilical vein endothelial cell (huvec) at 1–4% v/v (Chao et al., 2015), in adrenal gland pheochromocytoma in a concentration dependent manner (Yang et al., 2019), and in liver-injured mice at 0.25 and 0.5% diet (Yin et al., 2017); and that of *G. segetum* (IC_50_ for zymosan = 2.63 ± 0.89 μg/ml; IC_50_ for PMA = 1.41 ± 0.63 μg/ml) in polymorphonuclear cells (PMNs) neutrophils. The compound isolated from *G. segetum* extract, 8,8′-(ethene-1,2-diyl)-dinaphtalene-1,4,5-triol, possessed ROS inhibitory effect upon the activation by PMA (IC_50_ = 0.13 μM) and zymosan (IC_50_ = 0.05 μM). At the same time, rutin, which was also isolated from *G. segetum* extract, inhibited the ROS activated by PMA (IC_50_ = 0.08 μM) and zymosan (IC_50_ = 0.13 μM). Both isolated compounds from *G. segetum* showed a higher antioxidant effect than the positive control aspirin (Yuandani et al., 2017). One of the compounds isolated from *G. nepalensis* extract, 3,5-dicaffeoylquinic acid ethyl ester, showed an inhibitory effect on intracellular ROS production in cardiomyoblasts at a concentration of 0.78 μM and above (Yu et al., 2016). In nuclear and mitochondrial DNA, the interaction of the hydroxyl radical (HO•) with the nucleobases of the DNA strand will produce 8-hydroxy-2′-deoxyguanosine (8-OHdG), a predominant form of a free radical-induced oxidative lesion, which is a critical biomarker of oxidative stress (Valavanidis et al., 2009). *G. divaricata* diet (5 and 10%) showed the inhibition of the hepatic 8-OHdG level in a diabetic mice model (Dong et al., 2019). *G. procumbens* also improved the plasma TAS in rats with induced oxidative stress (Akowuah et al., 2012).

#### *Gynura* Inhibits Lipid Peroxidation

Lipid peroxidation is a reaction of oxygen with unsaturated lipids that successively results in the production of oxidation products, including lipid peroxyl radicals and hydroperoxides. Among a wide variety of oxidation products from lipid peroxidation, malondialdehyde (MDA) appears to be the most mutagenic product (Ayala et al., 2014). *G. procumbens*, *G. divaricata*, and *G. formosana* exhibited inhibition of lipid peroxidation by lowering the MDA level in carbon tetrachloride-induced oxidative stress rats (Akowuah et al., 2012), carcinogen-induced colon cancer rats (Shwter et al., 2014), high-fat-diet and streptozotocin (STZ)-induced diabetic mice (Xu et al., 2015; Dong et al., 2019), cotton pellet-induced granuloma rats (Ma et al., 2017), postmenopausal rats fed with cholesterol diet enriched with repeatedly heated palm oil (Nazri et al., 2019), and non-alcoholic steatohepatitis mice (Liu Y. et al., 2019). The reduction in the MDA level of *G. procumbens* extract-treated rats was comparable with treatments of anticancer drug, 5-fluorouracil (Shwter et al., 2014), and lipid-lowering drug atorvastatin (Nazri et al., 2019). It is worth noticing that Xu et al. (2015) and Dong et al. (2019) both studied on *G. divaricata* using identical treatment methods and diabetic mice models. The results of the study by Xu et al. (2015) showed a significant inhibitory effect of *G. divaricata* lyophilized powder on lipid peroxidation at treatment doses as low as 1.2% of diet. However, only 10% lyophilized powder diet was able to exert significant inhibition of lipid peroxidation in the study by Dong et al. (2019). This variation between findings could be due to the phytochemical or bioactivity variations that possibly correlated to intrinsic factors (age of the plant and part of the plant used) and extrinsic factors (geographical climate, nature of soil, season, and processing methods).

#### *Gynura* Modulates Enzymatic Antioxidant Production or Activities

The antioxidant mechanism of heme oxygenase-1 (HO-1) is found to be associated with an increase in superoxide dismutase (SOD) and CAT (Turkseven et al., 2005). HO-1 is responsible for the oxidative cleavage of heme groups, which generates biliverdin, carbon monoxide, and ferrous iron. Biliverdin is converted to bilirubin, and both of these bile pigments are potent scavengers of singlet oxygen (Stocker et al., 1990). Oral administration of *G. procumbens* extract was shown to increase HO-1 activity in liver-injured mice model (Liu Y. et al., 2019). SOD is an antioxidant enzyme that is responsible for the catalytical conversion of the superoxide radical (^∗^O_2_) or singlet oxygen radical (^1^O_2_^−^) to hydrogen peroxide (H_2_O_2_) and molecular oxygen (O_2_). However, the accumulation of H_2_O_2_ causes toxicity to body tissues or cells. Successively, CAT breaks down H_2_O_2_ into water and molecular oxygen, thus minimizing free radical-induced damage (Ighodaro and Akinloye, 2018). *G. procumbens*, *G. divaricata*, and *G. formosana* showed an antioxidant effect by inducing *in vivo* total SOD activity (Shwter et al., 2014; Xu et al., 2015; Ma et al., 2017; Dong et al., 2019; Nazri et al., 2019). The *in vitro* CAT activity was induced by *G. bicolor* (Chao et al., 2015; Yang et al., 2019) and a compound isolated from *G. nepalensis*, which were 25 and 50 μM of 3,5-dicaffeoylquinic acid ethyl ester (Yu et al., 2016). At the same time, *in vivo* CAT activity was increased by *G. procumbens* (Liu Y. et al., 2019; Nazri et al., 2019), *G. bicolor* (Pai et al., 2019), and *G. formosana* (Ma et al., 2017). Notably, the induction of SOD and CAT by the *G. procumbens* extract was comparable to the effects of atorvastatin (Nazri et al., 2019).

#### *Gynura* Modulates Glutathione-Related Parameters

Owing to the absence of CAT in the mitochondria, the reduction of H_2_O_2_ and lipid peroxides is carried out by GSH peroxidase (GSH-Px). Therefore, protection of cells against oxidative stress is reinforced by increasing the activity of GSH-Px, which plays a crucial role in inhibiting the lipid peroxidation process (Ighodaro and Akinloye, 2018). GSH-Px activity was induced by *G. procumbens* (Liu Y. et al., 2019; Nazri et al., 2019), *G. bicolor* (Chao et al., 2015; Yin et al., 2017; Pai et al., 2019; Yang et al., 2019), and *G. divaricata* (Xu et al., 2015; Dong et al., 2019). *G. procumbens* showed higher efficacy in inducing GSH-Px activity as compared to atorvastatin (Nazri et al., 2019). GSH is a crucial low-molecular-weight antioxidant that protects cells from oxidative damage via reduction, conjugation, and interaction with other non-enzymatic antioxidants (Forman et al., 2009). *G. formosana* extract diet (250 and 500 mg/kg) was found to increase hepatic GSH level in granuloma rat model (Ma et al., 2017), and *G. bicolor* extract also showed *in vitro* and *in vivo* preservation on GSH content (Chao et al., 2015; Yin et al., 2017; Pai et al., 2019; Yang et al., 2019). GSH reductase (GR) is vital in maintaining the supply of reduced GSH, and GSH disulfide (GSSG) indicates the level of oxidized GSH (Chakravarthi et al., 2006). *G. bicolor* extract (0.25, 0.5, and 1% of diet) maintained GR activity (Yin et al., 2017; Pai et al., 2019) and suppressed the hepatic GSSG content in the liver-injured mice model (Yin et al., 2017). GSH-*S*-transferase (GST) exerted protection on cellular macromolecules from damage of reactive electrophiles by catalyzing the conjugation of GSH to various endogenous and exogenous electrophilic compounds (Townsend and Tew, 2003). *G. procumbens* extract diet showed antioxidant potential by increasing *in vivo* GST activity (Shwter et al., 2014).

### Anti-inflammatory Effects of Genus *Gynura*

Table 4 shows the studies on the anti-inflammatory effects of *Gynura*. The potential mechanisms for the anti-inflammatory activity of *Gynura* are suggested as follows: modulation of inflammatory cytokine production, inhibition of PGE_2_ and NO production, inhibition of cellular inflammatory-related parameters, and inhibition of inflammation on animal models. As shown in Figure 2, the several potential anti-inflammatory signaling pathways of *Gynura* are PI3K/Akt, Nrf2, PPARγ, GSK3, NF-κB, and MAPK.

**TABLE 4:** List of studies on the anti-inflammatory effects of genus *Gynura.*

Plant species	Part, *Gynura* form	Cell line/Animal study model	Concentration/dose, control groups	Parameter measured and technique used	Findings	Reference
Gynura procumbens (Lour.) Merr.	Leaf	Human HaCaT keratinocytes	100, 500 μg/ml (24 h)	IL-6 and IL-8 production level using ELISA	By 100 and 500 μg/ml GP extract, IL-6 and IL-8 were inhibited.	Kim et al. (2011)
	Extract (Ethanol)	Human dermal fibroblasts (HDFs)	1, 10, 20 μg/ml (48 h)	MMP-1 expression using western blotting		
		*In vitro* study stimulated by UV irradiation	Control groups:	MMP-9 expression using Zymography	GP extract dose-dependently inhibited MMP-1 and MMP-9 expression in UV-B irradiated HDFs.	
			Normal control			
			Model control (UV 40 mJ/cm^2^)			
			Positive control (10 μM Retinoic acid)			
	Leaf, stem	RAW 264.7 macrophages	GPEO: 0.003, 0.01, 0.03 μg/ml	Inhibition on inflammatory cell infiltrates using migration assay	GPEO ↓↓↓ LPS-induced cell migration. Limonene, but not α-pinene, 3-carene, or their components mixture, ↓↓↓ cell migration.	Huang et al. (2019)
	Essential oil and its active ingredients	*In vitro* study stimulated by LPS	Active ingredients: concentration not stated			
			Treatment time not stated			
			Control groups:			
			Normal control			
			Model control (LPS)			
	Extract (Ethanol)	*In vitro* study stimulated by LPS	0.4, 0.6, 0.8 mg/ml	TNF-α and IL-6 production level using ELISA	GP extract dose-dependently ↓ IL-6. GP extract at 0.8 mg/ml ↓ TNF-α. Ethyl acetate fraction showed the best inhibitory effect on IL-6 and TNF-α.	Liu M. et al. (2019)
	Fractions (Petroleum ether, ethyl acetate, n-butanol, water)		24 h incubation with LPS			
			Control groups:			
			Normal control			
			Model control (1 μg/ml LPS)			
	Whole plant	RAW 264.7 macrophages	3.9, 15.63, 62.5 and 250 μg/ml	Nitric oxide production level using Griess assay	Pre-treatment of 250 μg/ml GP:	Ning et al. (2019)
	Extract (Ethanol)	*In vitro* study stimulated by LPS	1 h pre-treatment	iNOS protein expression using western blotting	↓ NO production (dose dependent)	
			Control groups:		↓ iNOS protein expression.	
			Normal control			
			Vehicle control (0.1% DMSO)			
			Model control (1 μg/ml LPS)			
	Leaf	Mice normal liver cell line NCTC-1469	80 and 160 μg/ml (24 h)	Nrf2 and *p*-JNK protein expressions using western blotting	80 μg/ml GP treatment ↑ Nrf2 protein level, ↓ *p*-JNK	Liu Y. et al. (2019)
	Extract (Aqueous)	*In vitro* study transfected with Ad-shCFLAR	Control groups:		160 μg/ml GP treatment ↑ Nrf2 protein levels, ↓↓ *p*-JNK	
			Normal control (Ad-shCtrl)			
			Model control (pre-treated with Ad-shCFLAR for 24 h)			
	Leaf	RAW 264.7 macrophages	15.63, 31.25, 62.5, 125, 250, 500 μg/ml	Nitric oxide production level using Griess assay	GP extract showed anti-inflammatory activity by inhibiting NO production	Chandradevan et al. (2020)
	Extract (Ethanol)	*In vitro* study stimulated by LPS	Control groups:			
			Normal control			
			Model control (IFN-γ + LPS)			
	Aerial part	Balb/c white mice (5 mice/group)	0.75 mg/20 μl	Ear thickness (anti-inflammatory activity)	Original organic crude extract ↓↓↓ croton oil-induced ear inflammation.	Iskander et al. (2002)
	Extract (Ethanol)	*In vivo* study using croton oil-induced ear inflammation mice model	Topical application			
			30 min pre-treatment, ear thickness was measured at 24 h			
			Control groups:			
			Model control (20 μl acetone at left ear + 0.1 mg/20 μl/ear croton oil)			
			Standard anti-inflammatory agent (hydrocortisone)			
	Leaf	Male BALB/c mice (5 mice/group)	50 mg/kg body weight	GSK3β phosphorylation using western blotting	GP ↑ phosphorylation of liver GSK3β (Ser9) compared with non-treated control	Wong et al. (2015)
	Extract (Ethanol)	*In vivo* study using parasite-infected mice model	Intraperitoneally treated	TNF-α, IFN-γ and IL-10 production level using ELISA	↓ TNF-α and IFN-γ levels in liver and serum by administration of GP	
			1 day pre-infection		↑ IL-10 level in serum.	
			Control groups:			
			Normal control			
			Model control (B. pseudomallei infection)			
	Leaf, stem	50 male Kunming mice (5 mice/group)	GPEO: 0.433, 0.865, 1.73 mg/ml	Ear thickness by xylene-induced ear edema	GPEO ↓↓ xylene-induced ear and hind paw edema at all doses throughout experiment. Treatment with active ingredients mixture ↓ ear and hind paw edema.	Huang et al. (2019)
	Essential oil and its active ingredients	*In vivo* study using xylene and formalin-induced inflammation mice model	Active ingredients: α-pinene (0.174 mg/ml), 3-carene (0.153 mg/ml), limonene (0.036 mg/ml) and mixture of all 3 ingredients	Hind paw edema using micrometre and histological examination		
			Topical administration	COX-2 expression using immunohistochemical staining and multispectral imaging analysis	↓ COX-2 expression by 0.865 mg/ml GPEO and all 3 individual and mixture of active ingredients.	
			Control groups:		↓↓ COX-2 expression by 1.73 mg/ml GPEO.	
			Untreated control			
			Model control (topical application of sesame oil + 20 μl xylene, intradermal injection of 20 μl 4% formalin)			
			Positive control (diclofenac diethylamine emulgel (DDE))			
	Leaf	32 Male C57BL/6J mice (8 mice/group)	500 and 1,000 mg/kg body weight	Nrf2 and *p*-JNK protein expressions using western blotting	500 mg/kg GP treatment ↑ Nrf2 protein level, no obvious effect on *p*-JNK, ↑ PPARγ mRNA expression.	Liu Y. et al. (2019)
	Extract (Aqueous)	*In vivo* study using non-alcoholic steatohepatitis (NASH) mic	Oral route	mRNA expression of PPARγ using RT-qPCR		
			Daily for 6 weeks		1000 mg/kg GP treatment ↑ protein levels of hepatic Nrf2, ↓ protein level of hepatic *p*-JNK, ↑↑ PPARγ mRNA expression.	
			Control groups:			
			Normal control (methionine- and choline-sufficient (MCS) diet)			
			Model control (methionine- and choline-deficient (MCD) diet)			
Gynura bicolor (Roxb. ex Willd.) DC	Leaf	RAW 264.7 macrophages	15, 30, 60, or 120 μg/ml	Nitric oxide production level using Griess assay	↓ NO production (30% decrease with 120 μg/ml GB)	Wu et al. (2013)
	Extract (Ether)	*In vitro* study stimulated by LPS	3 h pre-treatment	PGE2 production using competitive enzyme immunoassay (EIA) kit	↓ PGE2 production (dose dependent)	
			Control groups:	iNOS, COX-2, phosphorylated-IκBα and p65 protein expression using immunoblot analysis	↓ iNOS and COX-2 protein expressions (concentration dependent)	
			Vehicle control group (0.1% (v/v) methanol)	NF-κB DNA-binding activity by Nuclear protein preparation and electrophoretic mobility shift assay (EMSA	↓ *p*-IκBα protein (47% decrease with 120 μg/ml GB)	
			Model control (1 μg/ml LPS)		↓ nucleic p65 protein levels (3–41% decrease with 30, 60, 120 μg/ml GB)	
					↓ translocation of NF-κB from the cytosol to nuclei	
					↓ DNA-binding activity of NF-κB nuclear protein by 30, 60, 120 μg/ml GB	
	Leaf	Human umbilical vein endothelial cells (HUVEC)	Aqueous or ethanol extract at 1, 2 or 4% (v/v)	IL-6 and TNF-α production using cytoscreen immunoassay	Pre-treatments with aqueous or ethanol extract of GB at 2 and 4%	Chao et al. (2015)
	Extract (Aqueous, Ethanol)	*In vitro* study treated by high glucose	12 h pre-treatment	Prostaglandin E (PGE2) production	↓ IL-6, TNF-α formation	
			Control groups:	Cyclooxygenase (COX)-2 activity	↓ PGE2 formation	
			Control (5.5 mM glucose)		↓ COX-2 activity.	
			Model control (33 mM glucose)			
	Leaf	PC12 cell line (rat adrenal gland pheochromocytoma)	Aqueous extract at 0.25, 0.5 or 1%	LDH activity	↓ LDH activity in concentration dependent manner	Yang et al. (2019)
	Extract (Aqueous)	*In vitro* study of H2O2 induced injury	48 h pre-treatment	Mitochondrial membrane potential (Δψm) using fluorescent dye Rh123	↑ Δψm in concentration dependent manner	
			Control groups:	IL-6, IL-1β and TNF-α production level by cytoscreen assay	↓ IL-6, IL-1β and TNF-α production level	
			Normal control	NF-κB and p38 mRNA expression using RT-PCR	Test concentrations (0.25, 0.5 and 1%) ↓ NF-κB mRNA expression	
			Model control (H2O2 stimulation)		0.5 and 1% extract ↓ p38 mRNA expression.	
	Leaf	Male C57BL/6 mice (8 mice/group)	0.25 or 0.5% G. bicolor aqueous extract diet	Hepatic levels of IL-1β, IL-6 and TNF-α using cytoscreen immunoassay kits	GB dose-dependently ↓ hepatic levels of inflammatory cytokines of IL-1β, IL-6 and TNF-α.	Yin et al. (2017)
	Extract (Aqueous)	*In vivo* study using chronic ethanol consumption-induced hepatic injury mice model	Oral route			
			6 weeks			
			Control groups:			
			Normal control			
			Liquid diet group (without ethanol)			
			Ethanol diet group			
	Leaf	50 male Balb/cA mice (10 mice/group)	0.25, 0.5, 1% GB diet	Cardiac or renal level of IL-1β, IL-6 or TNF-α using cytoscreen immunoassay kits	GB at 3 doses:	Pai et al. (2019)
	Extract (Aqueous)	*In vivo* Streptozotocin-induced type 1 diabetic mice model	Oral route	p38 and NF-κB mRNA expression using RT-PCR	↓ level of IL-1β, IL-6, and TNF-α in heart and kidney	
			8 weeks		↓ p38 and NF-κB mRNA expression in heart or kidney	
			Control groups:			
			Normal control (basal diet)			
			Diabetic model control (40 mg/kg BW streptozotocin (in citrate buffer, 0.1 M) via i.p. injection for 5 days)			
*Gynura pseudochina* (L.) DC.	Leaf	HeLa cells	0.2–200 μg/ml	IL-6/luciferase assay (NF-κB assay)	*Gynura pseudochina* var. hispida (MeOH) showed the strongest NF-κB inhibitory effects, as well as inhibition on release of IL-1, IL-6, TNF-α and PGE2.	Siriwatanametanon et al. (2010)
	Extract (Methanol, Ethyl acetate, Petroleum ether)	*In vitro* study stimulated by PMA	1, 10 and 50 μg/ml	IL-6, IL-1, TNF-α and PGE2 production level using ELISA and EIA		
		Monocytes from healthy human donors	24 h			
		*In vitro* study stimulated by LPS	Control groups:			
			Positive control (ethanol)			
			Negative control (Unstimulated cells)			
			Reference group (Parthenolide, hydrocortisone)			
	Leaf	HeLa cells	Non-toxic concentrations (using the MTT assay)	IL-6/luciferase assay (NF-κB assay)	Quercetin 3-rutinoside showed the highest NF-κB inhibitory effect.	Siriwatanametanon and Heinrich (2011)
	Isolated compound from methanol extract	*In vitro* study stimulated by PMA	Control groups:		NF-κB inhibitory activities IC50:	
			Positive control (ethanol)		Quercetin 3-rutinoside: 24.1 ± 0.1 μg/ml	
			Negative control (Unstimulated cells)		3,5-di-caffeoylquinic acid: 42.8 ± 0.2 μg/ml	
			Reference group (Parthenolide)		4,5-di-caffeoylquinic acid: 49.1 ± 0.1 μg/ml	
					5-mono-caffeoylquinic acid: 83.0 ± 0.1 μg/ml	
	Leaf	Human HaCaT keratinocytes	Extract: 375 and 750 μg/ml	IL-8 production level using ELISA	Extract at both tested concentrations and some concentration of each marker compounds:	Sukadeetad et al. (2018)
	Extract (Ethanol), marker compounds	*In vitro* study stimulated by TNF-α	Chlorogenic acid: 140 and 280 μg/ml	RelA and RelB localization by immunofluorescence assay	↓ IL-8 production level	
			Caffeic acid: 30 and 60 μg/ml		↓ translocation of RelB S573 into nucleus	
			Rutin: 750 and 1,500 μg/ml			
			*p*-coumaric acid: 1,400 and 2,800 μg/ml			
			24 h			
			Control groups:			
			Normal control			
			Model control (50 ng/ml TNF-α with/without 0.7% DMSO)			
			Positive control (50 μg/ml curcumin)			
	Leaf	25 patients with mild to moderate plaque psoriasis	Mixture of extract and vehicle (1:10)	Phosphorylation of NF-κB p65 using immunohistochemistry (skin sections from two patients)	Immunohistochemical staining revealed diminution of phosphorylated NF-κB p65 in the lesions treated with the GP ointment.	Rerknimitr et al. (2016)
	Ointment from ethanol extract	Randomized controlled study	Twice daily for 4 weeks			
			Control: 0.1% triamcinolone cream			
*Gynura divaricata* (L.) DC.	Leaf, stem	60 male imprinting control region mice (15 mice/group)	Diets with 1.2 and 4.8% GD	Pancreatic Akt, PI3K, and PDK-1 mRNA expressions using qPCR	1.2% GD:	Xu et al. (2015)
	Lyophilized into powder	*In vivo* study using high-fat diet and streptozotocin (STZ) induced type 2 diabetic mice	Oral route	Pancreatic *p*-Akt, PI3K, and PDK-1 protein expressions using western blotting	↑ Akt mRNA, ↑ *p*-Akt protein expression, ↑ PI3K mRNA and protein expression, ↑ PDK-1 mRNA and protein expression	
			Daily for 4 weeks			
			Control groups:		4.8% GD:	
			Normal control (Normal diet)		↑↑ Akt mRNA, ↑↑ *p*-Akt protein expression, ↑↑ PI3K mRNA and protein expression, ↑↑ PDK-1 mRNA, ↑ PDK-1 protein expression.	
			Diabetic model control (high-fat diet (18% lard, 20% sugar, 3% egg yolk, 59% basal diet) and 100 mg/kg STZ)			
	Aerial part	Male ICR mice (15 mice/group)	Diets with 1, 5 and 10% GD	Western blotting to determine hepatic protein expression of	1% GD: ↑ *p*-GSK3β, ↑ PPARγ	Dong et al. (2019)
	Lyophilized into powder	*In vivo* study using high-fat diet and streptozotocin (STZ) induced type 2 diabetic mice	Oral route	phosphatidylinositol 3-kinase (PI3K)		
			Daily for 4 weeks	phosphorylated protein kinase B (*p*-Akt)		
			Control groups:	phosphorylated glycogen synthase kinase 3β (*p*-GSK3β)	5% GD: ↑ PI3K, ↑ *p*-Akt, ↑ *p*-GSK3β, ↑ PPARγ, ↓ TNF-α	
			Normal control (Normal chow)	PPARγ		
			Model control (high-fat diet (18% lard, 20% sugar, 3% egg yolk and 59% basal diet) and 100 mg/kg STZ)	Tumor necrosis factor-α (TNF-α)	10% GD: ↑ PI3K, ↑↑ *p*-Akt, ↑ *p*-GSK3β, ↑↑ PPARγ, ↓ TNF-α, ↓ NF-κB	
				Nuclear factor kappa B (NF-κB)		
*Gynura* segetum (Lour.) Merr	Leaf	RAW 264.7 macrophages	Extract: 6.25–100 μg/ml	Nitric oxide production level using Griess assay	GS extract showed inhibition on NO production with IC50 = 0.16 ± 0.03 μg/ml 8,8′-(ethene-1,2-diyl)-dinaphtalene-1,4,5-triol depicted the strongest NO inhibitory activity with IC50 value of 0.15 μM.	Yuandani et al. (2017)
	Extract (Methanol), isolated compound from extract	Peripheral blood mononuclear cells (PBMCs)	Compounds: 3.125–50 μg/ml	IL-1β and TNF-α production level using ELISA	GS extract showed IC50 TNF-α = 16.20 ± 3.94 μg/ml; IC50 IL-1β = 2.72 ± 1.84 μg/ml 4,5,4′-trihydroxychalcone demonstrated the highest inhibition on IL-1β release (IC50 = 6.69 μM). Rutin was the most potent sample against TNF-α release with IC50 = 16.96 μM.	Seow et al. (2014)
		*In vitro* study induced by LPS (1 μg/ml)	3 h (Griess assay) or 12 h pre-treatment (ELISA)			
			Control groups:			
			Positive control (0.025 μM Dexamethasone)			
		Male Sprague-Dawley rats	125, 250 and 500 mg/kg body weight	Cotton pellet granuloma assay	GS dose-dependently ↓↓↓ formation of granuloma tissues (17.1, 39.7, and 47.2% inhibition by 125, 250 and 500 mg/kg GS), ↓↓↓ TNF-α and IL-1 levels in circulating pro-inflammatory cytokine levels.	
	Extract (Methanol)	*In vivo* study using cotton pellet-induced granuloma rat model	Oral route	TNF-α and IL-1 production level using ELISA		
			Once daily for 7 days			
			Control groups:			
			Negative control (1% Tween 80)			
			Reference group (5 mg/kg indomethacin)			
*Gynura* nepalensis DC.	Leaf	H9c2 cardiomyoblasts	1.56, 3.12, 6.25, 12.5, 25, 50, 100 μM	LDH production level	Compound 6 (3,5-dicaffeoylquinic acid ethyl ester) exhibited a more potent cytoprotective effect thus selected for further evaluation.	Yu et al. (2016)
	Nine caffeoylquinic acid analogs (1–9) isolated from ethanol extract	*In vitro* study stimulated by H2O2	1 h pre-treatment	Mitochondrial membrane potential (Δψm)	↓ LDH leakage at 6.25, 12.5, 25, 50 and 100 μM compound 6.	
			Control groups:	Phosphorylation of ERK, JNK, and p38	↑↑↑ Δψm in cells cultured with compound 6 (6.25, 12.5, 25.0 μM).	
			Normal control		↓ Phosphorylation of JNK and ERK by 12.5 and 25.0 μM compound 6	
			Model control (0.3 mM H2O2)			
			Positive control (Carbonyl cyanide *m*-chlorophenylhydrazone (CCCP) for Δψm)			
	Leaf	24 Swiss albino mice (6 mice/group)	250 and 500 mg/kg body weight	Xylene-induced ear edema test	250 and 500 mg/kg GN extract ↓ xylene-induced ear edema and carrageenan-induced models of inflammation.	Rahman et al. (2018)
	Extract (Ethanol)	*In vivo* study using mice model with induced inflammation	Oral route	Carrageenan-induced paw edema test		
			1 h before infection			
			Control groups:			
			Model control (distilled water + 20 μl xylene, saline + injection of 0.1 ml 1% carrageenan)			
			Positive control (100 mg/kg diclofenac sodium)			
*Gynura formosana* Kitam.	Leaf	48 male Sprague-Dawley rats (8 rats/group)	100 mg/kg, 250 mg/kg, 500 mg/kg body weight	Cotton pellet granuloma assay	GF dose-dependently ↓ granuloma formation.	Ma et al. (2017)
	Extract (Ethyl acetate)	*In vivo* study using cotton pellet-induced granuloma rat model	Oral route	LDH, GPT, CRP, TNF-α and IL-1β production level using ELISA	100, 250, and 500 mg/kg GF ↓ levels of plasma inflammatory biomarkers (LDH, GPT and CRP) activities.	
			Once daily for 7 days			
			Control groups:		GF dose-dependently ↓ plasma pro-inflammatory cytokines (TNF-α and IL-1β).	
			Normal control			
			Model control (0.5% carboxymethylcellulose, 1 ml/kg)			
			Standard drug (4 mg/kg indomethacin)			

↑ indicates significantly induce (*p* < 0.05), ↑↑ indicates significantly induce (*p* < 0.01), ↑↑↑ indicates significantly induce (*p* < 0.001), ↓ indicates significantly inhibit (*p* < 0.05), ↓↓ indicates significantly inhibit (*p* < 0.01), and ↓↓↓ indicates significantly inhibit (*p* < 0.001).

#### *Gynura* Modulates Inflammatory Cytokine Production

Cytokines are released by cells for signaling, where critical pro-inflammatory cytokines, including IL-1, IL-6, IL-8, IFN-γ, and TNF-α, are involved in the upregulation of inflammatory reactions (Turner et al., 2014) and IL-10 is a potent anti-inflammatory cytokine (Zhang and An, 2007). *G. procumbens* inhibited the IL-6 and IL-8 production in UV-induced HaCaT keratinocytes (Kim et al., 2011). *G. procumbens* extract also dose-dependently suppressed the IL-6 production in macrophages and inhibited the TNF-α level. At the same time, the ethyl acetate fraction of *G. procumbens* showed the best inhibitory effect on IL-6 and TNF-α (Liu M. et al., 2019). In a parasite-infected mice model, *G. procumbens* reduced the levels of the liver and serum TNF-α and IFN-γ as well as increased IL-10 level (Wong et al., 2015). *G. bicolor* possessed an anti-inflammatory effect by inhibiting the production of IL-1β, IL-6, and TNF-α both *in vitro* (Chao et al., 2015; Yang et al., 2019) and *in vivo* (Yin et al., 2017; Pai et al., 2019). *G. pseudochina var. hispida* methanol extract also caused inhibition of IL-1β production (IC_50_ = 2.46 μg/ml). Meanwhile, ethyl acetate extract of *G. pseudochina var. hispida* showed strong inhibition of the release of IL-6 (IC_50_ = 8.14 μg/ml) and TNF-α (IC_50_ = 1.49 μg/ml) in monocytes (Siriwatanametanon et al., 2010). The extract of *G. pseudochina* and its marker compounds of chlorogenic acid, caffeic acid, rutin, and *p-*coumaric acid also showed significant inhibition of IL-8 production in keratinocytes (Sukadeetad et al., 2018). *G. segetum* extract inhibited the release of TNF-α (IC_50_ = 16.20 ± 3.94 μg/ml) and IL-1β (IC_50_ = 2.72 ± 1.84 μg/ml) in macrophages. Among the isolated compounds of *G. segetum*, 4,5,4′-trihydroxychalcone was the most potent sample in inhibiting IL-1β (IC_50_ = 6.69 μM), and another isolated compound, rutin, demonstrated the strongest inhibition against TNF-α release in macrophages (IC_50_ = 16.96 μM) (Yuandani et al., 2017). The anti-inflammatory effects of *G. segetum* and *G. formosana* have been demonstrated by the *in vivo* inhibition of TNF-α and IL-1, where the inhibitory effects of 500 mg/kg of *G. segetum* and *G. formosana* extracts were comparable to that of the nonsteroidal anti-inflammatory drug indomethacin (Seow et al., 2014; Ma et al., 2017). *G. divaricata* also suppressed TNF-α production in a diabetic mice model (Dong et al., 2019).

#### *Gynura* Inhibits Prostaglandin E_2_ and Nitric Oxide Production

Prostaglandin E_2_ (PGE_2_) is a bioactive lipid that physiologically mediates the regulation of immune responses, blood pressure, gastrointestinal integrity, and fertility. However, the sequential actions of cyclooxygenase-2 (COX-2) catalyze PGE_2_ synthesis. Consequently, dysregulated PGE_2_ production has been correlated to a variety of pathological conditions, such as chronic inflammation (Legler et al., 2010). Inhibition of COX-2 expression in mice model was exerted by *G. procumbens* essential oil and its active ingredients. Both *G. procumbens* essential oil and its active ingredients were showing similar COX-2 inhibitory effects as that of the positive control drug diclofenac diethylamine emulgel (Huang et al., 2019). The use of the *G. bicolor* extract inhibited PGE_2_ production and COX-2 protein expression and activity in activated macrophages (Wu et al., 2013) and endothelial cells (Chao et al., 2015). PGE_2_ production was also inhibited using the extract of *G. pseudochina var. hispida* with an IC_50_ value of 25.23 μg/ml (Siriwatanametanon et al., 2010).

Nitric oxide (NO) is a signaling molecule that physiologically exerts an anti-inflammatory effect and acts as a pro-inflammatory mediator in the overproduction of the enzyme iNOS (Sharma et al., 2007). *G. procumbens* extract had shown an inhibitory effect on NO production in macrophages (Liu M. et al., 2019; Ning et al., 2019; Chandradevan et al., 2020). This significant NO inhibitory effect by 250 μg/ml *G procumbens* was comparable to the positive control of the non-selective NOS inhibitor Nω-nitro-l-arginine methyl ester hydrochloride (L-NAME) (Ning et al., 2019). *G. procumbens* fractions (petroleum ether, ethyl acetate, *n-*butanol, and water) at concentrations of 0.3–0.8 mg/ml also exerted 2–64% inhibition of NO production in macrophages. Among the fractions, the ethyl acetate fraction possessed the highest NO inhibitory effect (Liu M. et al., 2019). The protein expression of iNOS in macrophages was shown to be inhibited by the *G. procumbens* extract at a concentration of 250 μg/ml (Ning et al., 2019). Similarly, *G. bicolor* showed concentration dependent suppression on iNOS protein expressions and NO production (30% decrease with 120 μg/ml extract) in macrophages (Wu et al., 2013). *G. segetum* extract also inhibited NO production in macrophages, with IC_50_ = 0.16 ± 0.03 μg/ml. Meanwhile, one of the compounds isolated from *G. segetum* extract, 8,8′-(ethene-1,2-diyl)-dinaphtalene-1,4,5-triol, depicted the strongest NO inhibitory activity with an IC_50_ value of 0.15 μM (Yuandani et al., 2017).

#### *Gynura* Inhibits Cellular Inflammatory-Related Parameters

Matrix metalloproteinases (MMPs) act as crucial regulatory enzymes in both pro- and anti-inflammatory pathways through the actions of cytokine or chemokine activation and antagonism (Manicone and McGuire, 2008). *G. procumbens* dose-dependently inhibited MMP-1 and MMP-9 expression in human dermal fibroblasts (HDFs), where the inhibition of the MMP-1 expression by the *G. procumbens* extract was even more effective than that of the positive control drug retinoic acid (Kim et al., 2011). Mitochondrial membrane potential (Δψm), the energy provider to generate ATP, is produced when free energy is used to pump protons out of the mitochondrial matrix via oxidative phosphorylation. Under a stress condition, Δψm can be reduced and can possibly lead to mitochondrial dysfunction in inflammatory responses (Yue and Yao, 2016). In a concentration dependent manner, Δψm was increased, and LDH activity was inhibited by *G. bicolor* extract (Yang et al., 2019) and the isolated compound from the *G. nepalensis*, 3,5-dicaffeoylquinic acid ethyl ester (Yu et al., 2016). The induction of Δψm by the isolated compound of *G. nepalensis* was more effective than that of carbonyl cyanide *m*-chlorophenyl hydrazone, a chemical inhibitor of oxidative phosphorylation (Yu et al., 2016). *G. formosana* also suppressed plasma inflammatory biomarker (LDH, GPT, and CRP) activities in the treated group, where these inhibitory effects were comparable to indomethacin (Ma et al., 2017). The essential oil of *G. procumbens* and its active ingredient, limonene, showed an inhibitory effect on inflammatory cell infiltration (Huang et al., 2019).

#### *Gynura* Inhibits Inflammation in Animal Models

Tissue swelling (edema) is one of the cardinal signs of inflammation, where the increased fluid filtration is further enhanced by the arteriolar vasodilator action of the inflammatory mediators (Amelang et al., 1981). *G. nepalensis* extract diet (Rahman et al., 2018), topical administration of *G. procumbens* extract (Iskander et al., 2002), *G. procumbens* essential oil, and the active ingredients mixture from the *G. procumbens* essential oil (Huang et al., 2019) showed anti-inflammatory effects by significantly reducing ear thickness and paw edema in inflammatory mice model. Granulomatous inflammation is a special variety of chronic inflammation in which cells of the mononuclear phagocyte system are aggregated into well-demarcated focal lesions called granulomas (Williams and Williams, 1983). The anti-inflammatory effect of *G. segetum* and *G. formosana* had been demonstrated by significant inhibition of granuloma tissue formation in a rat model (Seow et al., 2014; Ma et al., 2017).

#### Anti-inflammatory Signaling Pathways of *Gynura*

Mitogen-activated protein kinases (MAPK) consist of three signaling pathways: ERK, JNK, and p38 MAPK, which mediate fundamental cellular processes by regulating immunomodulatory cytokine expression (Kaminska, 2005). *G. bicolor* extract possessed an anti-inflammatory effect by inhibiting p38 mRNA expression both *in vitro* (Yang et al., 2019) and *in vivo* (Pai et al., 2019). The isolated compound from *G. nepalensis* extract, 3,5*-*dicaffeoylquinic acid ethyl ester, exhibited inhibitory effects on the phosphorylation of JNK and ERK in cardiomyoblasts (Yu et al., 2016). Similarly, *G. procumbens* treatment also inhibited the protein expression of p-JNK in murine hepatocytes and in a mice model (Liu Y. et al., 2019). NF-κB represents a family of inducible transcription factors that activate the transcription of various pro-inflammatory genes (Liu et al., 2017). The NF-κB signaling pathway had been proposed as a potential mechanism of the anti-inflammatory effect of *G. bicolor* through the inhibition of the p-IκBα protein, nucleic p65 protein levels, translocation of NF-κB from the cytosol to the nuclei, and DNA-binding activity of the NF-κB nuclear protein (Wu et al., 2013). To complement the study by Wu et al. (2013), two studies demonstrated that the *G. bicolor* aqueous extract was able to inhibit NF-κB mRNA expression in both *in vitro* (Yang et al., 2019) and *in vivo* studies (Pai et al., 2019). Meanwhile, *G. pseudochina var. hispida* extract caused the inhibition of NF-κB activation with IC_50_ = 41.96 μg/ml in HeLa cells (Siriwatanametanon et al., 2010). Successively, quercetin 3-rutinoside, which was one of the isolated compounds from *G. pseudochina var. hispida* extract, showed a strong NF-κB inhibitory effect with IC_50_ = 24.1 ± 0.1 μg/ml (Siriwatanametanon and Heinrich, 2011). *G. pseudochina* extract and its marker compounds of chlorogenic acid, caffeic acid, rutin, and *p-*coumaric acid also inhibited RelB S573 (protein transcription factors of NF-κB family) translocation into the nucleus of keratinocytes (Sukadeetad et al., 2018). Moreover, *G. divaricata* lyophilized powder at 10% of diet inhibited the NF-κB signaling pathway (Dong et al., 2019). In a randomized controlled study, NF-κB phosphorylation in the lesions treated with *G. pseudochina* ointment was inhibited (Rerknimitr et al., 2016).

Upon cellular induction, phosphoinositide 3-kinases (PI3K) generate lipid products that recruit cytosolic protein kinase B (Akt) to cellular membranes for Akt activation. Phosphoinositide-dependent kinase-1 (PDK-1) is a serine/threonine kinase that facilitates Akt phosphorylation. The activated Akt detaches from the plasma membrane and translocates through the cytosol to the nucleus for further downstream reactions (Vanhaesebroeck and Alessi, 2000). *G. divaricata* lyophilized powder in the diet of a diabetic mice model significantly induced the mRNA and protein expression of Akt and PI3K (Xu et al., 2015; Dong et al., 2019). Similarly, a mice model with *G. divaricata* lyophilized powder in its diet also exhibited significant induction of the mRNA and protein expression of PDK-1 (Xu et al., 2015). By Akt-mediated phosphorylation, the PI3K/Akt signaling pathway was able to inactivate GSK3 (Salazar et al., 2006), where GSK3 has been determined as one of the mechanisms that phosphorylate and degrade Nrf2. In turn, Nrf2, a transcription factor, will be upregulated and will ultimately lead to the production of downstream cytoprotective protein expression (Cuadrado et al., 2018). GSK3 acts as the central regulator of the inflammatory response to bacterial infections and other insults. Hence, inactivation of GSK3 by the phosphorylation of GSK3β has been proposed as a potential therapeutic target in the control of bacterial-driven inflammatory diseases (Wang et al., 2014). The anti-inflammatory effect of *G. procumbens* was correlated to the increased phosphorylation of liver GSK3β (Ser9), which inhibited the activities of GSK3 (Wong et al., 2015). *G. divaricata* lyophilized powder diet increased GSK3β phosphorylation in a diabetic mice model (Dong et al., 2019). *G. procumbens* treatment showed anti-inflammatory potential through the induction of the Nrf2 protein level in murine hepatocytes and in a mice model (Liu Y. et al., 2019). Akt activation is also essential for the transcriptional activation of peroxisome proliferator-activated receptor-γ (PPARγ) (Kim et al., 2010), a nuclear receptor that inhibits the expression of inflammatory cytokines and directs the differentiation of immune cells toward anti-inflammatory phenotypes (Tyagi et al., 2011). *G. procumbens* extract in diet significantly induced *in vivo* PPARγ mRNA expression (Liu Y. et al., 2019), and *G. divaricata* lyophilized powder diet increased *in vivo* hepatic protein expression of PPARγ (Dong et al., 2019).

#### Interplay Between Physiological, Biochemical and Immunological Aspects

The antioxidant and anti-inflammatory effects of *Gynura* species are corroborated to each other. As aforementioned, inflammation and oxidative stress are highly interdependent pathophysiological events. Hence the antioxidant effects of *Gynura* species has supported anti-inflammatory effects by these plants. In general, *Gynura* species depict anti-inflammatory effects by inhibiting inflammatory signaling pathways, cellular pathogenicity, inflammatory biomolecules secretion, and clinical manifestation of inflammatory diseases. Granulomatous inflammation is the end result of a prolonged complex interplay among causal agents, mononuclear phagocytes activity, circulating immune complexes, and a vast array of biological mediators (Zumla and James, 1996). Inflammatory cell infiltration is the critical process in granuloma formation while tissue swelling is one of the possible associated clinical manifestation. The pathogenesis of granulomatous inflammation is manipulated by the vital player of macrophages along with secretion of cytokines and chemokines by immune cells through stimulation of inflammatory signaling pathways (Facco et al., 2007). Not only granulomatous inflammation, the signaling pathways, biomolecules and cellular responses also contribute to pathogenesis of various diseases. Thus the plants in genus *Gynura* are having high potential to be explored for pharmacological evidence of different ailments.

## Future Perspective

Previous studies on *Gynura* species have indicated that several *Gynura* species possessed strong antioxidant and anti-inflammatory effects that are fundamental to various therapeutic purposes. Hence, *Gynura* species are potential continual source of new and useful bioactive compounds. Identification of the bioactive compounds in the plants contributing to the bioactivities, along with their mechanisms of action responsible for the pharmacological activities, is needed. Table 5 shows the list of phytochemicals identified in *Gynura* species. There is a need to investigate whether the crude extracts or isolated pure compounds are more effective. Synergistic effects of multiple active compounds in the extract may lead to a stronger pharmacological effect than that achievable by a single compound. At this stage, limited clinical studies on the antioxidant and anti-inflammatory effects of genus *Gynura* have been conducted. Human clinical trials with clearly defined symptomology to evaluate the therapeutic value of genus *Gynura* are necessary because animal experiments cannot be a substitute for clinical trials in the evaluation of therapeutic efficacy. To the best of our knowledge, there is only one non-systematic randomized controlled trial that has been carried out. Rerknimitr et al. (2016) investigated the efficacy of *G. pseudochina* DC. var. *hispida* Thv. ointment in treating chronic plaque psoriasis in a randomized controlled trial.

**TABLE 5:** Phytochemicals of *Gynura* species.

Species	Identified phytochemicals	References
*Gynura procumbens* (Lour.) Merr.	15,16-Dihydroxy-9Z, 12Z-octadecadienoic acid (PubChem CID: 16061068)	Akowuah et al. (2002), Rosidah et al. (2008), Kim et al. (2011), Kaewseejan and Siriamornpun (2015), Murugesu et al. (2017), Li et al. (2018), Murugaiyah et al. (2018), Nazri et al. (2019), Huang et al. (2019), Liu M. et al. (2019), Liu Y. et al. (2019), Chandradevan et al. (2020)
	3,4-Dicaffeoylquinic acid (Isochlorogenic acid B) (PubChem CID: 5281780)	
	3,5-Dicaffeoylquinic acid (Isochlorogenic acid A) (PubChem CID: 6474310)	
	3,5-O-Dicaffeoylquinic acid (PubChem CID: 13604688)	
	3-O-Methyl gallic acid sulfate (PubChem CID: not found)	
	3-Carene (PubChem CID: 26049)	
	4,5-Dicaffeoylquinic acid (Isochlorogenic acids C) (PubChem CID: 6474309)	
	4-O-Methyl gallic acid sulfate (PubChem CID: not found)	
	5-O-(E)-Caffeoyl-galactaric acid (PubChem CID: not found)	
	Apigenin (PubChem CID: 5280443)	
	Caffeic acid (PubChem CID: 689043)	
	Chlorogenic acid (PubChem CID: 1794427)	
	Choline (PubChem CID: 305)	
	Citric acid (PubChem CID: 311)	
	Cynarine (PubChem CID: 5281769)	
	Dicaffeoylquinic acids (PubChem CID: 6474310)	
	Eriocitrin (PubChem CID: 83489)	
	Ferulic acid (PubChem CID: 445858)	
	Feruloylquinic acid (PubChem CID: 10133609)	
	Gallic acid (PubChem CID: 370)	
	Genkwanin isomer (PubChem CID: 5281617)	
	Isobioquercetin (PubChem CID: not found)	
	Kaempferol (PubChem CID: 5280863)	
	Kaempferol 3-O-glucoside (Astragalin) (PubChem CID: 5282102)	
	Kaempferol 3-O-rhamnosyl-(1→6)-glucoside (PubChem CID: not found)	
	Kaempferol-3-O-rutinoside (Nicotiflorin) (PubChem CID: 5318767)	
	Limonene (PubChem CID: 22311)	
	Malic acid (PubChem CID: 525)	
	Myricetin (PubChem CID: 5281672)	
	Neochlorogenic acid (PubChem CID: 5280633)	
	Oxooctadecanoic acid (PubChem CID: 439332)	
	*p*-Coumaric acid (PubChem CID: 637542)	
	*p*-Coumaroylquinic acid (PubChem CID: 6441280)	
	*p*-Hydroxybenzoic acid (PubChem CID: 135)	
	Phenylalanine (PubChem CID: 6140)	
	Protocatechuic acid (PubChem CID: 72)	
	Quercetin (PubChem CID: 5280343)	
	Quercetin 3-O-rhamnosyl-(1→2)-galactoside (PubChem CID: 44259099)	
	Quercetin 3-O-rhamnosyl-(1→6)-glucoside (PubChem CID: not found)	
	Quercetin 3-O-rutinoside (Rutin) (PubChem CID: 5280805)	
	Sinapic acid (PubChem CID: 637775)	
	Syringic acid (PubChem CID: 10742)	
	Trimethyl gallic acid glucuronide (PubChem CID: not found)	
	Vanillic acid (PubChem CID: 8468)	
	α-Pinene (PubChem CID: 6654)	
*Gynura bicolor* (Roxb. ex Willd.) DC.	3,5-Di-O-caffeoylquinic acid (Isochlorogenic acid A) (PubChem CID: 6474310)	Lu et al. (2012), Wu et al. (2015), Qiu X. L. et al. (2018)
	3-O-Feruloylquinic acid (PubChem CID: 9799386)	
	3-O-*p*-Coumaroylquinic acid (PubChem CID: 9945785)	
	4,5-Di-O-Caffeoylquinic acid (Isochlorogenic acid C) (PubChem CID: 6474309)	
	5-O-Caffeoylquinic acid (Neochlorogenic Acid) (PubChem CID: 5280633)	
	5-O-*p*-Coumaroylquinic acid (PubChem CID: 9945785)	
	Anthocyanin (PubChem CID: 145858)	
	Caffeoyl glucose (PubChem CID: 129715972)	
	Citric acid (PubChem CID: 311)	
	Dihydro-phellopterin (PubChem CID: not found)	
	Gallic acid (PubChem CID: 370)	
	Geniposide (PubChem CID: 107848)	
	Guanosine (PubChem CID: 135398635)	
	Isobavachalcone (PubChem CID: 5281255)	
	Kaempferol-3-O-caffeoylate (PubChem CID: not found)	
	Kaempferol-3-O-glucoside (Astragalin) (PubChem CID: 5282102)	
	Malic acid (PubChem CID: 525)	
	Phenylalanine (PubChem CID: 6140)	
	Protocatechuate-O-glucoside (PubChem CID: not found)	
	Quercetin (PubChem CID: 5280343)	
	Quercetin-3-acetylhexose (PubChem CID: not found)	
	Quercetin-3-O-galactoside (PubChem CID: 5281643)	
	Quercetin-3-O-rutinoside (Rutin) (PubChem CID: 5280805)	
	Tryptophan (PubChem CID: 6305)	
	Uridine (PubChem CID: 6029)	
	β-Carotene (PubChem CID: 5280489)	
*Gynura pseudochina* (L.) DC.	(+)-Tephropurpurin (PubChem CID: 10047971)	Siriwatanametanon and Heinrich (2011), Ferlinahayati et al. (2017), Sukadeetad et al. (2018)
	1-(9Z-octadecenoyl)-sn-glycero-2,3-cyclic phosphate (PubChem CID: 52922109)	
	1,3,8-Trihydroxy-4-methyl-2,7-diprenylxanthone (PubChem CID: 67261902)	
	2-(2,4-Dihydroxyphenyl)-5-hydroxy-8-methyl-8-(4-methyl-3-penten-1-yl)-2,3-dihydro-4H,8H-pyrano[2,3-f]chromen-4-one (PubChem CID: not found)	
	3,4-Dihydroxycinnamoyl-(Z)-2-(3,4-Dihydroxyphenyl) Ethanol (PubChem CID: 14353342)	
	3,5-Dicaffeoyl quinic acid (Isochlorogenic acid A) (PubChem CID: 6474310)	
	3-O-Caffeoyl-1-O-methylquinic acid (PubChem CID: 131752768)	
	4,5-Dicaffeoyl quinic acid (PubChem CID: 13887346)	
	5-Hydroxy-2′-methoxy-6,7-methylenedioxyisoflavone (PubChem CID: 5491929)	
	5-Caffeoyl quinic acid (Chlorogenic acid) (PubChem CID: 1794427)	
	Caffeic acid (PubChem CID: 689043)	
	Isochlorogenic acid B (PubChem CID: 5281780)	
	Isochlorogenic acid C (PubChem CID: 6474309)	
	Kaempferol rutinoside (Nicotiflorin) (PubChem CID: 5318767)	
	Quercetin (PubChem CID: 5280343)	
	Quercetin 3-rutinoside (Rutin) (PubChem CID: 5280805)	
	Stigmasterol (PubChem CID: 5280794)	
	β-Sitosterol (PubChem CID: 222284)	
*Gynura divaricata* (L.) DC.	3,4-Dicaffeoylquinic acid (Isochlorogenic acid B) (PubChem CID: 5281780)	Jiangseubchatveera et al. (2015), Dong et al. (2019)
	3,5-Dicaffeoylquinic acid (Isochlorogenic acid A) (PubChem CID: 6474310)	
	4,5-Dicaffeoylquinic acid (Isochlorogenic acid C) (PubChem CID: 6474309)	
	3-Caffeoylquinic acid (Chlorogenic acid) (PubChem CID: 1794427)	
	Cubenol (PubChem CID: 519857)	
	Spathulenol (PubChem CID: 92231)	
*Gynura segetum* (Lour.) Merr.	Rutin (PubChem CID: 5280805)	Yuandani et al. (2017)
	Gallic acid (PubChem CID: 370)	
	4,5,4′-Trihydroxychalcone (PubChem CID: 468135)	
	8,8'-(Ethene-1,2-diyl)-dinaphtalene-1,4,5-triol (PubChem CID: not found)	
*Gynura nepalensis* DC.	3,4-Dicaffeoylquinic acid methyl ester (PubChem CID: not found)	Yu et al. (2016), Aktar et al. (2019)
	3,5-Dicaffeoylquinic acid ethyl ester (PubChem CID: not found)	
	3,5-Dicaffeoylquinic acid methyl ester (PubChem CID: 10075681)	
	3-O-cis-*p*-Coumaroylquinic acid (PubChem CID: 9945785)	
	4,5-Dicaffeoylquinic acid methyl ester (PubChem CID: not found)	
	Chlorogenic acid (PubChem CID: 1794427)	
	Isochlorogenic acid A (PubChem CID: 6474310)	
	Isochlorogenic acid B (PubChem CID: 5281780)	
	Isochlorogenic acid C (PubChem CID: 6474309)	
	Saponins (PubChem CID: 6540709)	
	Tannins (PubChem CID: 250395)	

The bioavailability of bioactive molecules from genus *Gynura* also has limited investigation. On the basis of the study by Wu et al. (2015), the use of *G. bicolor* extract showed improvement on *in vivo* iron absorption and storage protein, which might be related to its rich phytoactive ingredients. Although the traditional uses of genus *Gynura* are supported by scientific evidence, the processing, and application or consumption methods by the public may alter the phytochemical profile, thus leading to pharmacological effect variations. Herbal combination is possible to provide better effects or benefits in developing therapeutic drugs. The study by Sari et al. (2015) showed that the combination of *Andrographis paniculata* (Burm. f.) Ness and *G. procumbens* could be a potential candidate for the development of an antidiabetic agent. The optimum therapeutic effect could be attributed to the combination of the potent hypoglycemic effect of *A. paniculata* and the potent antioxidant effect of *G. procumbens*. Hence, further studies on the therapeutic effects of the combinations of *Gynura* species with other medicinal plants are potentially producing more effective disease remedies. However, the potential issues of herb–herb and herb–drug interactions should be given due consideration, and further studies are needed to ensure no adverse interactions in polypharmacy and polyherbacy conditions.

## Conclusion

The extracts and phytochemicals of several *Gynura* species, particularly *G. procumbens*, *G. bicolor*, *G segetum*, *G. divaricata*, *G. formosana*, *G. nepalensis*, and *G. pseudochina*, have been reported to exhibit strong antioxidant and anti-inflammatory effects. However, *in vitro* and *in vivo* studies and clinical studies that have been carried out on the species are by no means free of methodological flaws. All 27 studies selected in this systematic review depicted risk of bias at different extent. The lack of randomization, lack of blinding, and unclear methodology explanation are some prevalent limitations. Further preclinical studies, including toxicity and pharmacokinetic studies on *Gynura* extracts and their bioactive compounds, are necessary before they can be subjected to clinical studies. Genus *Gynura* has high potential to be developed into medicinal agents for prophylactic supplements of diseases related to oxidative stress or inflammation.

## Author Contributions

TJN, ZJ, and NMF designed the study. IJ and KH provided information on the ethnopharmacology of *Gynura* and sources of *Gynura* research. TJN conducted the literature search, extracted the data, and wrote the first draft. ZJ and NMF oversaw the research project, including checking the research work, reviewing, and interpreting the results. SMS and FB provided methodological advice on the project (literature search, screening, and selection). All authors are involved in reviewing and approval of the final manuscript.

## Funding

We would like to thank the Ministry of Higher Education for FRGS grant FRGS/1/2019/SKK06/UKM/02/1.

## Conflict of Interest

The authors declare that the research was conducted in the absence of any commercial or financial relationships that could be construed as a potential conflict of interest.

## Abbreviations

**Abbreviations:** ADI, acceptable daily intake; AOM, azoxymethane; BW, body weight; CAT, catalase; CCl_4_, carbon tetrachloride; COX-2, cyclooxygenase-2; CRP, c-reactive protein; DCFH-DA, 2′,7′-dichlorofluorescein diacetate; DMSO, dimethyl sulfoxide; EAE, enzyme-assisted extraction; EIA, enzyme immunoassay; ELISA, enzyme-linked immunosorbent assay; EMSA, electrophoretic mobility shift assay; ESI-MS, electrospray ionization-mass spectrometry; ESI-TOF-MS, electrospray ionization time-of-flight mass spectrometry; EThOS, e-theses online service; FRSA, free radical scavenging activity; GC-MS, gas chromatography-mass spectrometer; GPT, glutame pyruvate transaminase; GR, glutathione reductase; GSH, glutathione; GSH-Px, glutathione peroxidase; GSK3β, glycogen synthase kinase three beta; GSSG, glutathione disulfide; GST, glutathione-*S*-transferase; H, human; H_2_O_2_, hydrogen peroxide; HDFs, human dermal fibroblasts; HPLC, high-performance liquid chromatography; HP-TLC, high-performance thin-layer chromatography; HSOS, sinusoidal obstruction syndrome; huvec, human umbilical vein endothelial cell; HVOD, hepatic veno-occlusive disease; i. p., intraperitoneal; IC_50_, inhibitory concentration 50%; IFN-γ, interferon-gamma; IL, interleukin; iNOS, inducible nitric oxide synthase; LC-MS/MS, liquid chromatography with tandem mass spectrometry; LDH, lactate dehydrogenase; LPS, lipopolysaccharide; MDA, malondialdehyde; MeOH, methanol; MMP-1, matrix metalloproteinase-1; mRNA, messenger ribonucleic acid; NF-κB, nuclear factor kappa B; NGF, nerve growth factor; NMR, nuclear magnetic resonance; NO, nitric oxide; Nrf2, nuclear factor erythroid 2-related factor 2; OHAT, office health assessment and translation for conducting a systematic review and evidence integration; PBMCs, peripheral blood mononuclear cells; PGE_2_, prostaglandin E_2_; *p*-IκBα, phospho-IκB alpha; PMA, phorbol 12-myristate 13-acetate; PMNs, polymorphonuclear cells; PPARγ, peroxisome proliferator-activated receptor gamma; PRISMA, preferred reporting items for systematic reviews and meta-analyses; ROS, reactive oxygen species; RT-PCR, reverse transcription polymerase chain reaction; SIRT, sirtuin; SOD, superoxide dismutase; STZ, streptozotocin; TAS, total antioxidant status; TBARS, thiobarbituric acid reactive substances; TFC, total flavonoid content; TNF-α, tumor necrosis factor alpha; TPC, total phenolic content; UEAE, ultrasonic-enzyme-assisted extraction; UHPLC-Q-TOF-MS/MS, ultra-high-performance liquid chromatography-quadrupole time-of-flight mass spectrometry; UV, ultraviolet; v/v, volume/volume; v/w, volume/weight; Δψm, mitochondrial membrane potential.
